# Constrained Peptides with Fine‐Tuned Flexibility Inhibit NF‐Y Transcription Factor Assembly

**DOI:** 10.1002/anie.201907901

**Published:** 2019-10-17

**Authors:** Sadasivam Jeganathan, Mathias Wendt, Sebastian Kiehstaller, Diego Brancaccio, Arne Kuepper, Nicole Pospiech, Alfonso Carotenuto, Ettore Novellino, Sven Hennig, Tom N. Grossmann

**Affiliations:** ^1^ Chemical Genomics Centre of the Max Planck Society Otto-Hahn-Strasse 15 44227 Dortmund Germany; ^2^ Department of Chemistry and Pharmaceutical Sciences VU University Amsterdam De Boelelaan 1083 1081 HZ Amsterdam The Netherlands; ^3^ Department of Pharmacy University of Naples “Federico II” Via D. Montesano 49, 80131 Naples Italy

**Keywords:** constrained peptides, peptide inhibitors, protein structure, protein–DNA interactions, protein–protein interactions

## Abstract

Protein complex formation depends on the interplay between preorganization and flexibility of the binding epitopes involved. The design of epitope mimetics typically focuses on stabilizing a particular bioactive conformation, often without considering conformational dynamics, which limits the potential of peptidomimetics against challenging targets such as transcription factors. We developed a peptide‐derived inhibitor of the NF‐Y transcription factor by first constraining the conformation of an epitope through hydrocarbon stapling and then fine‐tuning its flexibility. In the initial set of constrained peptides, a single non‐interacting α‐methyl group was observed to have a detrimental effect on complex stability. Biophysical characterization revealed how this methyl group affects the conformation of the peptide in its bound state. Adaption of the methylation pattern resulted in a peptide that inhibits transcription factor assembly and subsequent recruitment to the target DNA.

## Graphical abstract

**Set Me free**: A peptide‐derived inhibitor of the NF‐Y transcription factor was developed by constraining the conformation of an epitope through hydrocarbon stapling and then fine‐tuning its flexibility. In the initial set of constrained peptides, a non‐interacting α‐methyl group was observed to have a detrimental effect on complex stability. Adaption of the methylation pattern gave a peptide that inhibits transcription factor assembly and subsequent DNA binding.

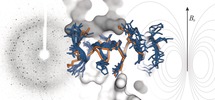

## Introduction

The assembly of proteins into multimeric complexes is central to many biological processes. The underlying protein–protein interactions (PPIs) involve a multitude of individual amino acid contacts and require the involved proteins to adopt a defined, but partially flexible, three‐dimensional structure. For selective and efficient protein assembly, the interplay between structural preorganization and flexibility is crucial, but it is often not clear how these parameters precisely influence complex stability. To investigate this interplay, isolated peptide motifs serve as valuable model systems. In this respect, α‐helices have drawn considerable attention since they represent a highly abundant secondary structure element in PPI interfaces.^1^ Short and isolated helical interaction motifs predominantly exist as flexible random coils when free in solution. Organization upon complex formation is associated with considerable entropic penalties and often results in low binding affinity. Preorganization of helical binding motifs can minimize these penalties and consequently lead to increased complex stability.1a

Tertiary structures in naturally folded proteins are stabilized by non‐covalent intramolecular interactions, the hydrophobic effect, and disulfide bridges. To compensate for the lack of such structural constraints in small helices, preorganization has been artificially achieved through backbone rigidification^2^ or macrocyclization strategies, including the formation of hydrogen‐bond surrogates^3^ and inter‐side‐chain crosslinks.1a, 4 The latter approach is often referred to as peptide stapling and can be implemented through a variety of crosslinking strategies, such as lactam formation,^5^ 1,3‐dipolar cycloaddition,4b, 6 thiol reactive ligation,^7^ and C−C bond formation.^8^ So‐called hydrocarbon stapled peptides^9^ combine two constraints: 1) backbone derivatization through amino acid α‐methylation^10^ and 2) the crosslinking of two alkene‐bearing side chains through ring‐closing metathesis.8a While the crosslink length has been extensively studied,^9^ the precise implications of the methyl group on peptide helicity and target binding are open questions.^11^ The hydrocarbon‐stapling approach has been used to stabilize α‐helical interaction motifs and has given rise to various bioactive PPI inhibitors of challenging protein targets, some of which were the first‐reported inhibitors for these targets.^12^

**Figure 1:**
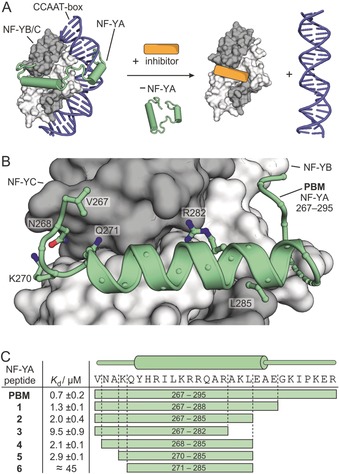
A) Schematic overview showing the NF‐Y trimer (NF‐YA: green, NF‐YB/C grey) bound to target DNA (blue). A potential inhibitor (orange) perturbs trimer assembly, thereby preventing NF‐Y from binding to DNA (structures based on PDB IDs: 4awl and 1nkp). B) Crystal structure (PDB ID: 6qmp, Table S2) of PBM (V267‐R295, green cartoon representation) in complex with the NF‐YB/C dimer (grey, surface representation). Selected PBM side chains are shown as ball‐and‐stick representation. For an overview of entire structure see Figure S2. C) The sequences of PBM (V267‐R295) and truncated peptides **1**–**6**, along with their dissociation constants (*K*_d_) as determined by FP (errors account for 1σ, measurements performed in triplicate; for binding curves see Figure S5).

Among PPIs, human transcription factor complexes represent particularly attractive therapeutic targets since many of them have implications in the onset and progression of certain forms of cancer.^13^ As often observed for PPI interfaces, the identification of potent inhibitors of transcription‐factor assembly is complicated by the large size of the involved interfaces and the lack of pronounced binding pockets. Even though constrained helical interaction motifs generally show a tendency to inhibit PPIs,1a only very few have been reported to directly target transcription factors with sufficient affinity to enable the inhibition of complex assembly.12a, 14 The nuclear transcription factor Y (NF‐Y)^15^ is a trimeric complex (NF‐YA/B/C) that binds to a particular DNA sequence (CCAAT box, Figure 1 A), thereby activating genes involved in regulation of the cell cycle and DNA repair.^16^ NF‐Y is considered a potential therapeutic target,^17^ but its direct and selective inhibition has proven to be challenging.^18^ Herein, we report the first structure‐based design of a stapled peptide that inhibits the assembly of NF‐Y. We altered the α‐methylation pattern of the involved non‐natural amino acids and were thus able to fine‐tune the flexibility of the peptide. This alteration results in increased affinity towards subunits of the transcription factor, thereby inhibiting its functional assembly as well as subsequent recruitment to the target DNA.

## Results and Discussion

### The 16‐mer NF‐YA Sequence is Crucial for NF‐YB/C Binding

NF‐Y subunits B and C form a stable heterodimer, which by itself does not possess relevant affinity to DNA target sequences (CCAAT box DNA). Only upon availability and binding of the third subunit (NF‐YA) is the transcriptionally active trimeric complex formed and DNA binding occurs with affinity values in the low nanomolar range.^19^ In this study, we aimed to inhibit the interaction between the NF‐YB/C heterodimer (light/dark grey) and NF‐YA (green) to prevent transcription‐factor assembly and DNA (blue) binding (Figure 1 A). The previously reported crystal structure of the NF‐Y trimer in complex with DNA (PDB ID: 4awl) reveals a 29‐mer NF‐YA sequence as the B/C binding motif (PBM, V267–R295).^20^ Aiming for the structural characterization of PBM bound to NF‐YB/C (B: aa 51‐143; C: aa 27‐120) in the absence of DNA, crystallization conditions were screened to provide crystals diffracting to 2.0 Å (space group *P*3_2_21). The resulting crystal structure (Figure 1 B, Figure S2, PDB ID: 6qmp) shows the NF‐YB/C dimer in the expected histone‐like fold, which superimposes closely with the corresponding domains in the DNA‐bound NF‐Y trimer (RMSD of 169 C_α_: 0.85 Å, PDB ID: 4awl, Figure S3). For PBM, we observe well‐defined electron density for all amino acids except G289 and R295, both located in the C‐terminal part of the peptide (Figure S2). The central part of PBM adopts an α‐helical conformation (Y272–A287), which is flanked by extended peptide stretches (Figure 1 B). Except for the C‐terminal part (K290–E294), PBM superimposes closely with NF‐YA in the DNA‐bound complex (RMSD for V267–E288: 0.52 Å, Figure S4).

To identify the crucial interaction motif within PBM, six truncated sequences (**1**–**6,** Figure 1 C) and PBM were synthesized and fluorescently labeled to determine dissociation constants with the NF‐YB/C dimer using a fluorescence polarization (FP) assay (Figure 1 C, Figure S5). PBM shows sub‐micromolar affinity for NF‐YB/C (*K*_d_=0.7±0.2 μm) and removal of seven (**1**) or ten (**2**) C‐terminal amino acids results in only moderate affinity losses (*K*_d_(**1**)=1.3±0.1 μm, *K*_d_(**2**)=2.0±0.4 μm). Further removal of three C‐terminal amino acids finally reduces binding considerably (*K*_d_(**3**)=9.5±0.4 μm). N‐terminal truncations were investigated using peptide **2** as a starting point. Removal of the first three amino acids causes only a minor affinity reduction (*K*_d_(**5**)=2.9±0.1 μm). Further N‐terminal truncation, however, abrogates NF‐YB/C binding almost completely (*K*_d_(**6**)≈45 μm), which determines the central α‐helix including a short N‐terminal segment as the core motif required for NF‐YB/C binding.

### Design of NF‐YA‐Derived Hydrocarbon‐Stapled Peptides

**Figure 2:**
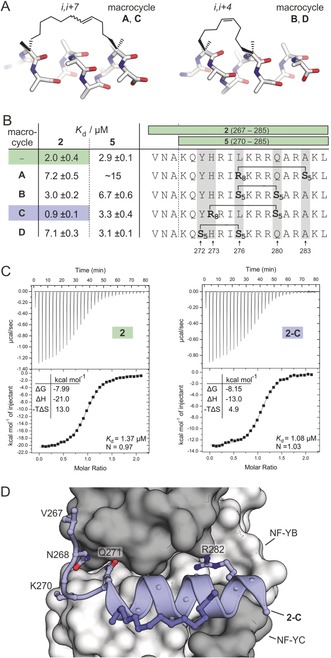
A) Model structures of hydrocarbon‐stapled peptides with *i*,*i*+7 (macrocycle **A**, **C**) and *i*,*i*+4 (macrocycle **B**, **D**) crosslinks. B) Sequences of modified peptides and their *K*_d_ values as determined by FP (errors account for 1σ, measurements performed as triplicates, binding curves are shown in Figure S5). C) Representative ITC curves of **2** and **2‐C** (measurements were performed in triplicate; for full data see Table S3 and Figure S8/ S9). D) Crystal Structure (PDB ID: 6qms, Table S2) of **2‐C** (blue cartoon representation) in complex with NF‐YB/C (grey surface representation). Selected **2‐C** side chains are shown as ball‐and‐stick representations.

Having identified the core binding motif of NF‐YA, we aimed for a stabilization of the central α‐helix to increase its affinity for the B/C dimer using the hydrocarbon‐stapling approach. The two required non‐natural α‐methylated and olefin‐bearing amino acids were introduced during solid‐phase peptide synthesis and subsequently crosslinked through ring‐closing olefin metathesis (Figure S6).^21^ Since hydrocarbon‐stapled peptides with *i*,*i*+7 and *i*,*i*+4 crosslinks (Figure 2 A) have been shown to have the highest degree of helicity,9b we decided to focus on these two architectures. Within the NF‐YA helix (Figure 1 B), we identified five solvent‐exposed amino acids (Y272, H273, L276, Q280, A283; Figure 2 B and Figure S7), which were used for crosslink incorporation. This resulted in four stapled helices, two with *i*,*i*+7 (macrocycle **A** and **C**) and two with *i*,*i*+4 (macrocycle **B** and **D**) crosslinks (Figure 2 A, B), all of which were introduced in two different lengths of the binding motif (16‐mer **5** and 19‐mer **2**, Figure 2 B). The resulting eight peptides were synthesized, and fluorescently labeled versions were used to determine dissociation constants with NF‐YB/C using FP (Figure 2 B, Figure S5). Compared to the linear peptides **2** and **5**, only the 19‐mer peptide harboring macrocycle **C** (**2‐C**) exhibits increased affinity for NF‐YB/C (*K*_d_(**2‐C**)=0.9±0.1 μm).

To compare the binding of unlabeled **2** and **2‐C**, we performed isothermal titration calorimetry (ITC; Figure 2 C, Table S3, Figure S8/S9), which shows a slightly increased NF‐YB/C affinity of stapled peptide **2‐C** (*K*_d_(**2‐C**)=1.08±0.06 μm) when compared to linear peptide **2** (*K*_d_(**2**)=1.37±0.03 μm). ITC measurements also confirm the expected 1:1 binding stoichiometry for peptide and NF‐YB/C. To investigate the binding mode, we co‐crystalized **2‐C** in complex with NF‐YB/C, which provided crystals that diffract to 1.8 Å (space group *P*2_1_2_1_2_1_). The resulting crystal structure of NF‐YB/C bound to **2‐C** (Figure 2 D, PDB ID: 6qms) shows a protein dimer overlaying closely with the one bound to PBM (RMSD=0.74 Å, Figure S10). Except for the C‐terminal amino acid L285, we observe well‐defined, continuous 2 *F*_o_−*F*_c_ electron density for stapled peptide **2‐C** that also includes the hydrocarbon crosslink (Figure S11). Superimposition of **2‐C** and corresponding residues in PBM shows a good overlap, thus confirming an analogous binding mode (RMSD=0.59 Å, Figure S12).

### Amino Acid α‐Methylation Determines Binding Affinity

**Figure 3:**
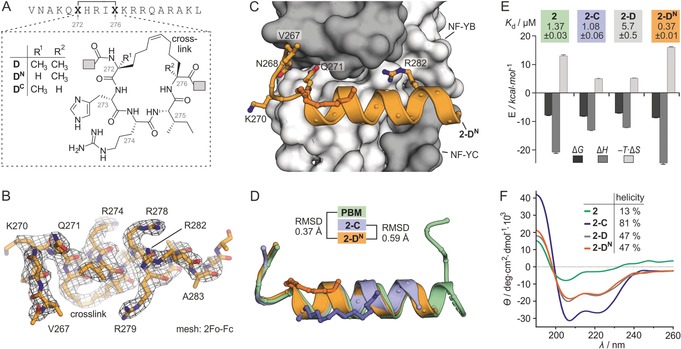
A) Chemical structures of macrocycles **D**, **D^N^** and **D^C^** as found in peptides **2‐D**, **2‐D^N^** and **2‐D^C^**. B) Electron‐density map (2 *F*_o_−*F*_c_, level 1σ) of **2‐D^N^** originating from the complex with NF‐YB/C (PDB ID: 6qmq, Table S2). C) Crystal structure (PDB ID: 6qmq) of **2‐D^N^** (orange cartoon representation) in complex with NF‐YB/C (grey surface representation). Selected **2‐D^N^** side chains are shown as ball‐and‐stick representations. D) Superimposition of the PBM, **2‐C**, and **2‐D^N^** originating from their complexes with NF‐YB/C (PDB IDs: 6qmp, 6qms and 6qmq; for overlays including protein surface, see Figure S14/15). E) Thermodynamic profile as determined by ITC, including *K*_d_ values (measurements were performed in triplicate, errors account for 1σ; for full data see Table S3 and Figure S8, S9, S16, S17). F) CD spectra in buffer (pH 7.4, *c*(peptide)=75 μm) and calculated helical content (for complete secondary‐structure distribution, see Table S4).

When comparing affinities of the two peptide lengths (16‐mer vs. 19‐mer) in our panel (Figure 2 B), we observed in four cases the expected trend: Shorter peptides (16‐mer: **5**, **5‐A**, **5‐B**, **5‐C**) exhibit lower affinity than their longer counterparts (19‐mer: **2**, **2‐A**, **2‐B**, **2‐C**). For macrocycle **D** though, the 16‐mer peptide (*K*_d_(**5‐D**)=3.1±0.1 μm) shows higher affinity than its 19‐mer analogue (*K*_d_(**2‐D**)=7.1±0.3 μm). Notably and in contract to the other crosslinks, macrocycle **D** replaces the most N‐terminal amino acid (Y272) in the α‐helix of the parent peptide PBM (Figure 1 C). Importantly, in all macrocycles, the two non‐natural and crosslinked amino acids (X) harbor an α‐methyl group, which influences the local peptide conformation, yet the precise implications for α‐helicity and binding affinity are unclear.^11^ For that reason, we were interested in whether the α‐methylation in macrocycle **D** causes the loss of affinity upon peptide elongation. Two derivatives of 19‐mer peptide **2‐D** were synthesized (Figure 3 A), one lacking α‐methylation at position 272 (**2‐D^N^**, N‐terminal X) and the other at position 276 (**2‐D^C^**, C‐terminal X). NF‐YB/C affinity for the fluorescently labeled versions was determined, which revealed slightly reduced affinity for peptide **2‐D^C^** (*K*_d_=9.9±0.8 μm), when compared to **2‐D** (*K*_d_=7.1±0.3 μm, Figure S5). In contrast, peptide **2‐D^N^** exhibits a more than 10‐fold increased affinity (*K*_d_(**2‐D^N^**)=0.70±0.04 μm). Importantly, upon truncation, this stapled peptide experiences the expected decrease in affinity (*K*_d_(**5‐D^N^**)=1.8±0.1 μm). Notably in the case of crosslinked peptide **2‐C** (*K*_d_=0.9±0.1 μm), the two derivatives lacking either the N‐ or C‐terminal α‐methyl group (**2‐C^N^** and **2‐C^C^**) show unchanged affinity for NF‐YB/C (*K*_d_=0.9±0.2 and 0.8±0.2 μm, Figure S5). The highest affinity peptide **2‐D^N^** was co‐crystalized with NF‐YB/C, yielding crystals diffracting up to 2.5 Å (space group *P*2_1_2_1_2_1_). The resulting crystal structure (PDB ID: 6qmq) shows the NF‐YB/C dimer in the expected fold (Figure S13). Peptide **2‐D^N^**, which includes the hydrocarbon crosslink, is clearly defined by the 2 *F*_o_−*F*_c_ electron density (Figure 3 B) and establishes protein contacts (Figure 3 C) also observed for **2‐C** and PBM. The overlay of **2‐D^N^**, **2‐C**, and PBM in their bound state (Figure 3 D) indicates an almost identical peptide conformation.

To obtain more insight into the characteristics of complex formation, ITC experiments for **2‐D** and **2‐D^N^** with NF‐YB/C were performed (Figure S16, 17). The binding stoichiometry of **2‐D^N^** (*N*=0.76) and **2‐D** (*N*=1.26) differs from the expected value (*N*=1), however, the binding affinity values are in line with FP data and reveal a 15‐fold higher affinity for peptide **2‐D^N^** (*K*_d_=0.37±0.01 μm) than for **2‐D** (*K*_d_=5.7±0.5 μm). Taken together this provides thermodynamic binding data for a total of four peptides (Figure 3 E and Table S3). Binding of unmodified peptide **2** is associated with a high entropic penalty (−*T*Δ*S*=13 kcal mol^−1^), which is compensated by a considerable binding enthalpy (Δ*H*=−21.0 kcal mol^−1^). Compared to peptide **2**, both peptides with a fully α‐methylated macrocycle (**2‐C** and **2‐D**) exhibit a reduced entropic penalty while the contributions by binding enthalpy are decreasing. Notably, **2‐D^N^** binding provides a thermodynamic profile similar to linear peptide **2**, and it shows the highest entropic penalty (−*T*Δ*S*=16.1 kcal mol^−1^) as well as the largest binding enthalpy (Δ*H*=−24.8 kcal mol^−1^) in our panel. To assess whether differences in binding originate from preorganization of the unbound peptides, we determined the α‐helicity of **2**, **2‐C**, **2‐D**, and **2‐D^N^** using circular dichroism (CD) spectroscopy (Figure 3 F and Table S4). Linear peptide **2** shows a very small helical content (13 %, green), while *i*,*i*+7 stapled peptide **2‐C** exhibits high α‐helicity (81 %, blue). For the two *i*,*i*+4 stapled peptides **2‐D** (grey) and **2‐D^N^** (orange), we observe very similar CD spectra indicating moderate helicity (47 %, Figure 3 F). This suggests that the altered α‐methylation pattern in these two peptides does not change their overall folding propensity.

### α‐Methylation Affects the Conformation of the Bound Peptide

It is surprising that peptide **2‐D^N^** binds NF‐YB/C with a 15‐fold higher affinity than **2‐D** although the only difference is the absence of a single methyl group that is not involved in direct contacts with the protein (Figure 3 C). This is particularly interesting considering their similar CD spectra. For this reason, we aimed for a more thorough investigation of their unbound states using ^1^H NMR spectroscopy in aqueous solution. Comparing Hα chemical shifts with random coil references again provides similar trends for the two peptides: Amino acids H273 to K277 show a clear deviation from random coil, thus indicating the presence of a defined secondary structure in the center of the free peptide, while the remaining N‐terminal (V267–Q271) and C‐terminal amino acids (R278–L285) appear to be relatively flexible (Figure S18). Overall, this is in line with their behavior in the CD measurements (47 % helicity for **2‐D** and **2‐D^N^**).

**Figure 4:**
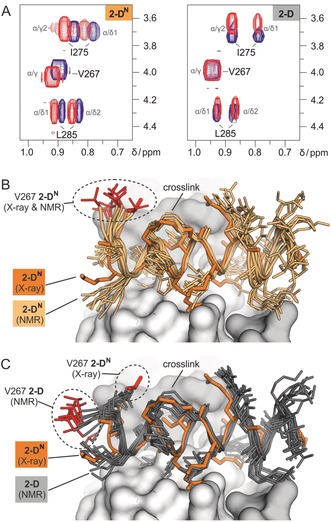
A) 2D ^1^H‐^1^ *H*‐TOCSY spectra of peptide **2‐D** and **2‐D^N^** in aqueous buffer in the presence (red) and absence (blue) of NF‐YB/C (for overview of involved residues groups, see Figure S18). B) Superposition of the crystal structure of **2‐D^N^** (orange, X‐ray) bound to NF‐YB/C (grey surface, PDB ID: 6qmq) and of the 10 lowest‐energy peptide conformers of **2‐D^N^** (light orange, NMR). Peptide backbones are shown as cartoon and side chains as stick models. The side chain of amino acid V267 is highlighted (red) in all structures. C) Superposition of the crystal structure of **2‐D^N^** (orange, X‐ray) bound to NF‐YB/C (grey surface, PDB ID: 6qmq) and of the 10 lowest energy peptide conformers of **2‐D** (dark grey, NMR). Peptide backbones are shown as cartoons and side chains as stick models. The side chain of amino acid V267 is highlighted (red) in all structures. Coordinates of the top ten NMR structures for bound **2‐D^N^** and **2‐D** are available as supporting material.

We hypothesized that the differences in NF‐YB/C‐binding originate from peptide characteristics in the bound state. Even though, we obtained a crystal structure for **2‐D^N^** bound to NF‐YB/C, it was not possible to crystallize **2‐D** in complex with NF‐YB/C. To assess potential differences between the bound states of **2‐D** and **2‐D^N^**, we performed 2D ^1^H‐^1^H TOCSY experiments with both peptides in the absence and presence of NF‐YB/C. For the higher‐affinity binder **2‐D^N^**, we observed chemical‐shift changes of well‐resolved methyl groups in V267, I275, and L285, thus indicating the involvement of all peptide regions (N‐terminus: V267, center: I275, C‐terminus: L285) in protein binding (Figure 4 A left). These observations are in line with the crystal structure of **2‐D^N^** in complex with NF‐YB/C, which shows a defined electron density for the entire peptide backbone (Figure 3 B). For the lower‐affinity peptide **2‐D**, TOCSY signals show chemical‐shift changes for I275 (central) and L285 (C‐terminus) upon NF‐YB/C binding. Importantly, the signal for V267 (N‐terminus) does not change upon binding (Figure 4 A right), thus indicating that the N‐terminal part of **2‐D** is not involved in direct contacts.

To obtain more details on the bound conformation of **2‐D** and **2‐D^N^**, transferred‐NOESY (tr‐NOESY) experiments were performed. tr‐NOESY provides structural information about a ligand in its bound state while analyzing resonances of the free ligand.^22^ NMR experiments were performed with an approximately 50‐fold excess of peptide over protein at a concentration that facilitates rapid exchange between free and bound peptides. NOEs indicative of the free state develop slowly. Thus, NOEs observed in the transferred NOE spectrum at short mixing times are indicative of the bound conformation of the peptide. Compared to their unbound state, both NF‐YB/C‐bound peptides show a few additional NOE correlation peaks and a considerable increase in the relative intensity of a number of NOE peaks, which is indicative for folded structures (Table S5). Using the tr‐NOEs of the bound peptides as input data for restrained simulated annealing calculations, ensembles of conformers of NF‐YB/C‐bound **2‐D** and **2‐D^N^** were obtained (Figures 4 B and C). When superimposed with its crystal structure (orange) in complex with NF‐YB/C, the NMR‐based conformers of bound **2‐D^N^** (light orange) show a very good overlay (Figure 4 B). Notably and in line with the TOCSY experiments, the N‐terminus of **2‐D** (grey, Figure 4 C) considerably deviates from the bound form **2‐D^N^** (orange) while the rest of the peptide overlays. Taken together, the results of the NMR and ITC experiments clearly show that the α‐methyl group at position 272 has a strong effect on peptide binding. This effect appears to be mainly caused by interference with the bound conformation of the peptide, since CD and NMR indicate very similar conformations for **2‐D** and **2‐D^N^** in their unbound state. Presumably, the bioactive conformation of the peptide observed in the crystal structures of **PBM**, **2‐C**, and **2‐D^N^** is perturbed by an α‐methyl group at position 272.

### Inhibition of DNA Binding through Disruption of NF‐Y Trimer Formation

**Figure 5:**
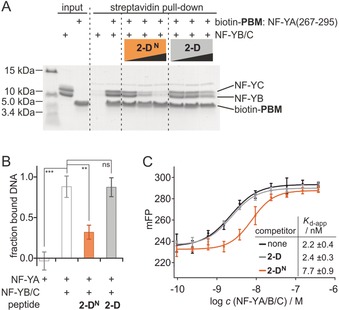
A) SDS PAGE gel of competition pull‐down. Bead‐immobilized biotinylated PBM incubated with NF‐YB/C (80 μm) in the presence or absence of **2‐D^N^** or **2‐D** (*c*=16, 80, 400 μm; for triplicate and full gels, see Figure S20). B) Fraction of bound DNA as determined by FP using TAMRA‐labeled DNA (*c*=5 nm) with NF‐YA(262–332) (*c*=25 nm) and NF‐YB/C (*c*=4 nm) in the absence and presence of peptide (*c*=10 μm, triplicate measurements, errors account for 1σ). ns: *p*>0.05, ** *p*<0.01, *** *p*<0.001. C) FP titration using TAMRA‐labeled DNA (*c*=5 nm) and varying concentrations of NF‐Y trimer (*c*=9.7×10^−11−^3.7×10^−7^ m) in the absence or presence of peptide (*c*=10 μm).

Having obtained high‐affinity peptide **2‐D^N^**, we tested its ability to interfere with the formation of the trimeric NF‐YA/B/C complex. For that purpose, competition pull‐down experiments were designed in which a biotinylated NF‐YA fragment (biotin‐PBM) was used to bind and immobilize the NF‐YB/C dimer. The pull‐down of NF‐YB/C was detected through SDS polyacrylamide gel electrophoresis (PAGE; Figure 5 A). Applying increasing concentrations of **2‐D^N^** (*c=*16–400 μm) results in a concentration‐dependent inhibition of complex formation between biotin‐PBM and NF‐YB/C (*c=*80 μm). As expected, the low‐affinity peptide **2‐D** exhibits only very weak inhibitory activity.

Given the ability of **2‐D^N^** to disrupt the NF‐Y trimer, we were interested in whether DNA binding of the NF‐Y trimer is also affected by this PPI inhibitor (Figure 1 A). To monitor the binding state of an NF‐Y target DNA containing the CCAAT‐box, a fluorescence polarization (FP)‐based assay was designed: Double‐stranded target DNA harboring a tetramethylrhodamine (TAMRA) label was incubated with the NF‐YB/C dimer and an NF‐YA fragment (aa 262–332), comprising the NF‐YB/C binding site and the DNA binding motif. FP measurements confirm the requirement of intact NF‐Y timer for DNA binding (88 % bound DNA, Figure 5 B). In the presence of peptide **2‐D^N^** (*c=*10 μm), we observe a considerable loss in the fraction of bound DNA (32 % bound DNA). In contrast when using peptide **2‐D**, no significant change in the DNA binding state is observed (87 % bound DNA). Finally, these findings were confirmed utilizing FP‐based titration experiments involving preformed NF‐Y trimer, which was titrated against the TAMRA‐labeled DNA (*K*_d_=2.2±0.4 nm, Figure 5 C). In the presence of **2‐D^N^**, the affinity between the NF‐Y trimer and the DNA deceases 3.5‐fold (*K*_*d*‐app_=7.7±0.9 nm), while peptide **2‐D** does not affect DNA binding by NF‐Y (*K*_*d*‐app_=2.4±0.3 nm).

## Conclusion

In this study, we show that peptide binding motifs can respond sensitively to slight changes in their structural preorganization. Fine‐tuning of their conformational properties can thus enable the design of inhibitors of challenging protein targets such as transcription factors. Initially, we identified a 14‐mer α‐helix with a short extended N‐terminal stretch as the protein fragment crucial for NF‐YB/C binding. To stabilize the α‐helix, a set of modified hydrocarbon‐stapled peptides was designed, thereby introducing an inter‐residue crosslink and an α‐methyl group at each of the two crosslinked amino acids. This set of modified peptides did not show a meaningful increase in binding affinity, thus indicating that solely focusing on the preorganization of the unbound peptide is insufficient. We observed an unusual binding behavior for one derivative (**2‐D**), where peptide shortening resulted in increased binding affinity. Knowing that amino acid α‐methylation can restrict the conformational freedom of the peptide backbone, we investigated how variation of the α‐methyl pattern influences binding affinity of **2‐D**. Only at position 272 did substitution of the methyl group by hydrogen affect binding, resulting in a peptide (**2‐D^N^**) with 15‐fold higher affinity.

It is surprising that such a small variation in a peptide region without direct protein contact results in a considerable affinity increase, in particular in relation to the size of the ligand (MW(**2‐D^N^**)=2351 g mol^−1^). To identify the cause of this difference, we investigated in detail both the free and the bound state of each peptide (**2‐D** and **2‐D^N^**). CD and NMR experiments indicated very similar conformational behavior in solution suggesting therefore differences in the bound state. For the higher‐affinity peptide (**2‐D^N^**), we obtained X‐ray and NMR structures in the NF‐YB/C‐bound form clearly showing contacts of the α‐helix and the extended N‐terminal stretch with the protein. For **2‐D** on the other hand, the NMR structure indicates that the additional α‐methyl group at position 272 induces an elongation of the α‐helical conformation towards the N‐terminus even when bound to the protein, resulting in loss of direct protein contacts. This observation is confirmed by ITC measurements, which show reduced binding enthalpy for **2‐D** compared with **2‐D^N^**. Therefore, we reason that specific release of conformational constraint by removal of the α‐methyl group allows adaption of the correct binding mode. Taken together, our study indicates that the stabilization of binding motifs composed of multiple secondary‐structure elements requires sections with relatively high conformational freedom to facilitate tertiary‐structure‐like folds. Given these intrinsic flexibility features and the complexity of the involved interaction areas, it is important to note that a fully rational affinity maturation of constrained binding epitopes is challenging. Consequently, the combination of structure‐based design with screening approaches applying focused peptide libraries^23^ could represent an appealing strategy.

## Conflict of interest

The authors declare no conflict of interest.

## Supporting information

As a service to our authors and readers, this journal provides supporting information supplied by the authors. Such materials are peer reviewed and may be re‐organized for online delivery, but are not copy‐edited or typeset. Technical support issues arising from supporting information (other than missing files) should be addressed to the authors.

Supplementary
