# Supplementary material for: Constrained Peptides with Fine‐Tuned Flexibility Inhibit NF‐Y Transcription Factor Assembly
Source: Angew Chem Int Ed Engl. 2019 Oct 17;58(48):17351–8. doi: 10.1002/anie.201907901 (PMC6900064; doi:10.1002/anie.201907901)
Supplement: Supplementary file 1 — Supplementary [file ANIE-58-17351-s001.pdf]

Angewandte Chemie
Eine Zeitschrift der Gesellschaft Deutscher Chemiker

Supporting Information

# Constrained Peptides with Fine-Tuned Flexibility Inhibit NF-Y Transcription Factor Assembly

*Sadasivam Jeganathan+, Mathias Wendt+, Sebastian Kiehstaller, Diego Brancaccio, Arne Kuepper, Nicole Pospiech, Alfonso Carotenuto, Ettore Novellino, Sven Hennig,* and Tom N. Grossmann**

anie_201907901_sm_miscellaneous_information.pdf

## Table of content

1. **Methods**......2

1.1. Solid-phase peptide synthesis......2

1.2. Plasmids and protein purification......3

1.3. Fluorescence polarization assays......4

1.4. Protein crystallization and structure determination......5

1.5. Isothermal titration calorimetry......5

1.6. Circular dichroism......6

1.7. Pull-down experiment......6

1.8. NMR spectroscopy......6

2. **Supplementary tables**......8

3. **Supplementary figures**......12

4. **References**......28

# 1. Methods

## 1.1. Solid-phase peptide synthesis

Peptides were synthesized using Fmoc-based solid-phase peptide synthesis.[1] All reaction steps were performed at room temperature in syringe reactors using Rink-amide ChemMatrix® resin. During reaction the resin was suspended on an orbital shaker. In between reaction steps the resin was washed with DMF (3×, 1 mL per 50 mg resin), DCM (3×, 1 mL per 50 mg resin) and DMF (3×, 1 mL per 50 mg resin) successively. Reagents and amino acids were purchased from Iris Biotech, Sigma Aldrich and Carl Roth. Unnatural amino acids were purchased from Okeanos Tech.

**Fmoc removal**

For the deprotection of the *N*-terminus the resin was suspended in a solution of Piperidine/DMF (2/8, v/v) 1 mL per 50 mg resin for 5 min. Subsequently the reaction solution was discarded and the reaction repeated.

**Amino acid coupling**

A solution of 4 eq. amino acid, 4 eq. COMU ((1-Cyano-2-ethoxy-2-oxoethyliden-aminooxy)dimethylamino-morpholino-carbenium hexafluorophosphate) and 4 eq. OXYMA (Ethyl cyano (hydroxyimino) acetate) in DMF was activated with 8 eq. of DIPEA (*N*,*N*-Diisopropylethylamine). Subsequently, the reaction solution was added to the resin (0.3 mL per 50 mg resin). After 20 min, the solution was discarded and the coupling repeated. For unnatural amino acids the reaction was performed once and the reaction time was extended to 1 h.

**Ring-closing metathesis (RCM)**

Prior reaction, the resin was washed with dry DCE (3×, 1 mL per 50 mg resin). The resin was suspended in a solution of Grubbs catalyst 1$^{st}$ generation (4 mg $mL^{-1}$) in dry DCE (1 mL per 50 mg resin). During reaction, a continuous flow of nitrogen was applied. After 2 hours, the solution was discarded and the reaction was repeated three times.

**Acetylation of *N*-terminus**

The *N*-terminal amino group was acetylated using 1 mL per 50 mg resin of $Ac_2O$/DIPEA/DMF (1/1/8, v/v/v). After 10 min, the reaction solution was discarded and the acetylation repeated. Afterwards, the resin was washed with DCM (3×, 1 mL per 50 mg resin).

***N*-terminal FITC labelling**

For the fluorescence polarization binding assay, peptides were *N*-terminal labelled with FITC (fluorescein isothiocyanate). Therefore, a PEG2 linker (Fmoc-PEG2-OH, 8-(9-Fluorenylmethyloxycarbonyl-amino)-3,6-dioxaoctanoic acid) was *N*-terminal linked using the amino acid coupling protocol. The Fmoc protecting group was removed and the resin was suspended in a solution of 6 eq. FITC and 12 eq. DIPEA in DMF (1 mL per 50 mg resin). After 16 h, the reaction solution was discarded and the resin was washed with DCM (3×, 1 mL per 50 mg resin).

**Cleavage, purification and characterization**

Peptides were cleaved using a solution of TFA/TIPS/$H_2O$ (95/2.5/2.5, v/v/v, 1 mL per 50 mg resin, 3 h). Afterwards, the TFA was evaporated and peptides were precipitated in diethyl ether. Precipitate was separated via centrifugation (15 min, 4 °C, 4000 rcf), the supernatant was removed and crude peptides were dissolved in ACN/$H_2O$ (1/1, v/v). Peptide purification was performed using reversed-phase HPLC (Column: Macherey-Nagel Nucleodur C18, 10 × 125 mm, 110 Å, 5 µm) with solvent A ($H_2O$ + 0.1 % TFA) and solvent B (ACN + 0.1 % TFA). For separation a gradient elution was used with 20 – 80 % of solvent B over 40 min and a flow rate of 6 mL $min^{-1}$. Subsequently, peptides were characterized using a reverse-phase HPLC coupled to ESI-MS (Column: Agilent Zorbax C18, 4.6 × 150 mm, 5 µm). Quantification was performed gravimetrically or via FITC absorption (λ = 495 nm; ε = 77000 $M^{-1}$ $cm^{-1}$) in 100 mM sodium phosphate buffer pH 8.5 (Table S1).

## 1.2. Plasmids and protein purification

**Plasmids**

The DNA fragments of NF-YB (containing amino acid region 51-143) and NF-YC (containing amino acid region 27-120) were inserted into pACYAduet (Novagen) vector using sequence and ligation independent cloning (SLIC) method.[2] The DNA fragment of NF-YA (containing amino acids region 262-332) was cloned into pGEX-6p-2 (GE Healthcare Life Sciences) using EcoRI restriction site. These are the confirmed protein sequences:

NF-YA (aa 262-332):

GPLGSPGILEEPLYVNAKQYHRILKRRQARAKLEAEGKIPKERRKYLHESRHRHAMARKRGEGGRF
FSPKEKDSPHMQDP

NF-YB (aa 51-143:):

GPSFREQDIYLPIANVARIMKNAIPQTGKIAKDAKECVQECVSEFISFITSEASERCHQEKRKTIN
GEDILFAMSTLGFDSYVEPLKLYLQKFRE

NF-YC (aa 27-120):

GPMEEIRNLTVKDFRVQELPLARIKKIMKLDEDVKMISAEAPVLFAKAAQIFITELTLRAWIHTED
NKRRTLQRNDIAMAITKFDQFDFLIDIVPR

**Protein purification**

Protein expression of NF-YB/C was performed in *E. coli* BL21(DE3). Expression culture was inoculated with overnight culture and incubated at 37 °C, till an $OD_{600}$ of 1 was reached. Subsequently, the culture was induced with 0.4 mM IPTG and incubated overnight at 22 °C. Cells were harvested via centrifugation (4000 rpm, 20 min, 4 °C, JA8.100 rotor Beckman Coulter). The cell pellet was suspended in lysis buffer (25 mM Tris pH 8.0, 1 M NaCl, 0.1 mM PMSF, 1 mM TCEP, DNase, Lysozyme). Cells were lysed via microfluidizer (LM10,

Microfluidics). Cell debris was removed by centrifugation (25000 rpm, 40 min, 4 °C, JA25.50 rotor Beckman Coulter). The supernatant was loaded onto 5 mL HisTrap FF Crude column (GE Healthcare) at a flow rate of 1 mL min$^{-1}$. The column was then washed with 10 column volume of wash buffer (25 mM Tris pH 8.0, 1 M NaCl, 0.1 mM PMSF, 1 mM TCEP, 40 mM Imidazole). The column bound protein was cleaved off by over-night incubation using PreScission protease in cleavage buffer (25 mM Tris pH 8.0, 400 mM NaCl, 40 mM Imidazole). Cleaved NF-YB/C protein was concentrated via ultra-filtration (Amicon ultra centrifugal filters, Millipore, MWCO: 10 kDa). Subsequent size exclusion chromatography (Superdex S75 16/60, GE Healthcare Life Sciences) was performed in SEC buffer (25 mM Tris pH 8.0, 1 M NaCl, 1 mM DTT). The pure fractions were pooled, concentrated via ultra-filtration and the final concentration was determined at 280 nm ($\varepsilon_{NF\text{-}YB/C}$ = 10095 $M^{-1}$ $cm^{-1}$, yield = 5.5 mg per L expression culture).

NF-YA expression was performed in *E. coli* BL21(DE3). Expression culture was inoculated with overnight culture and incubated at 37 °C, till a $OD_{600}$ of 1 was reached. Subsequently culture was induced with 0.4 mM IPTG and incubated overnight at 22 °C. Cells were harvested via centrifugation (4000 rpm, 20 min, 4 °C, JA8.100 rotor Beckman Coulter). The cell pellet was resuspended in lysis buffer (50 mM Tris pH 8.0, 400 mM NaCl, 2 mM $MgCl_2$, 1 mM EDTA, 5 mM β-mercaptoethanol, 0.1 mM PMSF, DNase, Lysozyme). Cells were lysed via microfludizer (LM10, Microfluidics). Cell debris was removed by centrifugation (25000 rpm, 40 min, 4 °C, JA25.50 rotor, Beckman Coulter). The supernatant was loaded onto a 20 mL glutathione sepharose column (GE Healthcare Life Science) at a flow rate of 1 mL min$^{-1}$. Bound protein was cleaved from the column by over-night incubation using PreScission protease. Subsequent size exclusion chromatography (Superdex S75 16/60 column, GE Healthcare Life Sciences) was performed in SEC buffer (25 mM HEPES, pH 8.0, 400 mM NaCl, 2 mM $MgCl_2$, 2 mM DTT). The pure fractions were pooled, concentrated via ultra-filtration and the final concentration was determined at 280 nm ($\varepsilon_{NF\text{-}YA}$ = 4470 $M^{-1}$ $cm^{-1}$, yield = 15 mg per L expression culture).

## 1.3. Fluorescence polarization assays

**Direct binding of peptides to heterodimer NF-YB/C**

NF-YB/C dimer was serially diluted in FP buffer (10 mM HEPES pH 7.4, 250 mM NaCl, 0.001 % Tween-20) on a 384-well plate (Corning: black, low volume, non-binding surface, round bottom). Peptide stocks in DMSO (100 µM) were diluted in FP-buffer to 40 nM and added to the serial dilution of NF-YB/C to a final concentration of 10 nM peptide in each well and $1.8\cdot10^{-4}$ – $7.1\cdot10^{-10}$ M NF-YB/C. After 1 h of incubation the fluorescence polarization values were measured at room temperature ($\lambda_{Ex}$ = 470 nm; $\lambda_{Em}$ = 525 nm). Measurements were performed in triplicates. The dissociation constants were determined using non-linear regression in Prism 5.0 (Graphpad).

**TAMRA-based FP measurements**

For DNA binding studies, a TAMRA labelled 25 bp long DNA fragment[3] derived from the HSP70 promotor was used.

Hsp70-fwd: TAMRA – 5′ TTCTGAG**CCAAT**CACCGAGCTCGAT 3′

Hsp70-rev: 5′ ATCGAGCTCGGTG**ATTGG**CTCAGAA 3′

The determination of fraction bound (fb) DNA is based on FP measurements ($\lambda_{Ex}$ = 530 nm; $\lambda_{Em}$ = 585 nm) at different concentrations of DNA, NF-YA, NF-YB/C and peptide. Free DNA (fb = 0) was measured in buffer 10 mM HEPES pH 7.4, 250 mM NaCl, 0.001 % Tween-20. These FP measurements were performed in presence of 5 nM TAMRA-labelled DNA (see above). The maximum FP value (fb = 1) was determined using 25 nM NF-YA and 60 nM NF-YB/C (plateau was confirmed by another measurement at 150 nM NF-YB/C resulting in similar FP values). Peptide inhibition was tested at 25 nM NF-YA and 4 nM NF-YB/C in the absence and presence of 10 µM peptide (**2-D** or **2-D$^{N}$**). All measurements were performed in triplicates.

For NF-YA/B/C trimer titration, equimolar amounts for NF-YB/C and NF-YA were pre-incubated (buffer: 10 mM HEPES pH 7.4, 250 mM NaCl, 0.001 % Tween-20). Subsequently FP titration experiments were performed with $9.7 \cdot 10^{-11}$ – $3.7 \cdot 10^{-7}$ M of NF-YA/B/C and 5 nM DNA in the absence and presence of 10 µM peptide. Measurements were performed in triplicates ($\lambda_{Ex}$ = 530 nm; $\lambda_{Em}$ = 585 nm).

## 1.4. Protein crystallization and structure determination

Samples for crystal trails were prepared by mixing the purified protein and peptide in a ratio of 1:2 – 1:4 at concentration of 5 – 10 g $L^{-1}$ in 25 mM Tris pH 8.0, 250 mM NaCl, 1 mM TCEP. The initial crystallization hits were obtained using sitting-drop 96-well (3-well) iQ-plates (TTP Labtech) using JSGC screens I-IV (Qiagen). Drops were set up using Mosquito (TTP Laptech) by mixing 100 nL protein/peptide mixture and 100 nL reservoir solution. The best initial hits were subsequently optimized and scaled-up in 24-well XRL-plates (Molecular Dimensions) by the hanging-drop vapor diffusion method using 1 µl protein/peptide mixture and 1 µl reservoir solution. Crystals of NF-YB/C/**PBM** were obtained at 0.1 M Tris pH 9.0, 0.2 M sodium acetate and 30 % PEG 4000. The crystals of NF-YB/C/**2-C** were grown at 0.1 M Tris pH 8.5, 0.2 M magnesium chloride and 18 % PEG 8000. The crystals of NF-YB/C/**2-D$^{N}$** were grown at 0.1 M sodium cacodylate pH 8.5, 0.2 M calcium acetate and 18 % PEG 600.

The diffraction data of cryo-cooled crystals (100 K) was collected at X10SA beamline at SLS (Villigen, Switzerland). The collected data sets were integrated using XDS[4]. The structures of NF-YB/C and peptide (PDB ID: 6qmp, 6qms, 6qmq) were solved with molecular replacement (Phaser)[5] using NF-YB/C (PDB ID: 4csr) as search model. Rounds of model building (Coot)[6] and refinement (phenix.refine of the PHENIX software suite)[7] resulted in the final model. Statistics for data collection and refinements are shown in Table S2.

## 1.5. Isothermal titration calorimetry

The ITC measurements were performed on a Malvern MicroCal Auto-iTC200 instrument. The concentration of NF-YB/C in the cell was adjusted to 50 µM in ITC buffer (25 mM Tris pH 8, 250 mM NaCl, 0.5 mM TCEP), the concentration of the peptide in the syringe was adjusted to 500 µM, also in ITC buffer. 30 injections per

measurements were performed at 25 °C (1.25 µl injection volume, 2 s injection time, 150 s spacing) with an initial delay of 60 s and a stirring speed of 750 rpm. Measurements were performed in triplicates (Figure S8, S9, S16, S17,). ITC curves were analysed using the programme OriginLab (Table S2).

### 1.6. Circular dichroism

CD spectra were obtained using the spectropolarimeter J-715 from Jasco. *N*-terminally FITC-labeled peptides were dissolved in 10 mM sodium phosphate buffer pH 7.4 (75 µM). For each peptide ten spectra from 260 nm to 190 nm were recorded in continuous scanning mode and averaged (sensitivity: 10 mdeg, resolution: 1.0 nm, response: 1.0 s, bandwidth: 1.0 nm, and scanning speed: 50 nm $min^{-1}$). Background was subtracted prior and the spectra were smoothed using a FFT filter. CD data are presented as mean residual ellipticity [Θ]. The ratio of secondary structure elements was calculated with CDNN using the PEPFIT[8] set of reference spectra for secondary structure determination (Table S4).

### 1.7. Pull-down experiments

For each pull-down sample Streptavidin coated beads (GE healthcare, loading capacity 300 nmol $mL^{-1}$) were equilibrated by washing (3×) with PD buffer (20 mM Tris, pH 7.5, 250 mM NaCl). Subsequently the beads were incubated (4 °C, 30 min) with 80 µM biotinylated **PBM** in PD buffer with a molar ratio of 1:1 (bt-**PBM**: bead binding sites). After washing with PD buffer (3×), the beads were incubated (4 °C, 1 h) with an equimolar amount of NF-YB/C in the absence and presence of acetylated peptide **2-D** and **2-D$^{N}$** (16 µM, 80 µM and 400 µM). For unspecific NF-YB/C binding to the Streptavidin coated beads protein was incubated with beads without prior **bt-PBM** loading. After incubation the beads were washed with PD buffer (3×) and suspended in elution buffer (25 mM Biotin) and SDS sample buffer (4:1, v/v). The NF-YB/C/**bt-PBM** complex was eluted using high temperature (95 °C, 10 min). Samples were analysed by SDS-PAGE. The experiment was performed in triplicates (Figure S20).

### 1.8. NMR spectroscopy

The samples for NMR spectroscopy were prepared by dissolving the appropriate amount of peptide in 0.2 mL of buffer (25 mM acetate-$D_4$ pH 5.5, 250 mM KCl, 10 % (v/v) deuterium oxide) to obtain a concentration of 1 mM. NMR spectra were recorded on a Varian INOVA 700 MHz spectrometer equipped with a z-gradient 5 mm triple-resonance probe head. All the spectra were recorded at a temperature of 25 °C. One-dimensional (1D) NMR spectra were recorded in the Fourier mode with quadrature detection. The water signal was suppressed by gradient echo.[9] 2D DQF-COSY,[10] TOCSY,[11] and NOESY[12] spectra were recorded in the phase-sensitive mode using the method from States.[13] Data block sizes were 2048 addresses in $t_2$ and 512 equidistant $t_1$ values. Before Fourier transformation, the time domain data matrices were multiplied by shifted $\sin^2$ functions in both dimensions. A mixing time of 80 ms was used for the TOCSY experiments. NOESY experiments were run with a mixing time of 100 ms. 2D tr-NOESY spectra were measured at mixing time of 100 ms using 1 mM of peptides and 20 µM protein (50-fold ligand excess) in buffer (25 mM acetate-D4 buffer at pH 5.5, containing 250 mM KCl, 10 % (v/v) deuterium oxide). The

qualitative and quantitative analyses of DQF-COSY, TOCSY, and NOESY spectra, were obtained using the interactive program package XEASY.[14] Coordinates of the top ten NMR structures for bound **2-D$^{N}$** and **2-D** are available as supporting material.

**Spectra analysis**

Almost complete $^{1}$H NMR chemical shift assignments were effectively achieved for **2-D** and **2-D$^{N}$** according to the Wüthrich[15] procedure via the usual systematic application of DQF-COSY, TOCSY, and NOESY experiments with the support of the XEASY software package.

**Peptide structure determination**

The NOE-based distance restraints were obtained from tr-NOESY spectra of **2-D** and **2-D$^{N}$**. The NOE cross peaks were integrated with the XEASY program and were converted into upper distance bounds using the CALIBA program incorporated into the program package DYANA.[16] Only NOE derived constraints were considered in the annealing procedures. NMR-derived upper bounds were imposed as semiparabolic penalty functions with force constants of 4 kcal mol$^{-1}$ Å$^{-2}$. A distance maximum force constant of 1000 kcal mol$^{-1}$ Å$^{-2}$ was used. Crystal structure of peptide **2-D$^{N}$** in complex with NF-YB/C were used as starting coordinates for both compounds. Atomic potentials and charges were assigned using the consistent valence force field (CVFF).[17] The conformational space of compounds was sampled through 100 cycles of restrained Simulated Annealing (ε = 1r). In Simulated Annealing, the temperature is altered in time increments from an initial temperature to a final temperature by adjusting the kinetic energy of the structure (by rescaling the velocities of the atoms). The following protocol was applied: the system was heated up to 1000 K over 2000 fs (time step = 1.0 fs); the temperature of 1000 K was applied to the system for 2000 fs (time step = 1.0 fs) with the aim of surmounting torsional barriers; successively, temperature was linearly reduced to 300 K in 1000 fs (time step = 1.0 fs). Resulting conformations were then subjected to restrained Molecular Mechanics (MM) energy minimization within Insight Discover module (ε = 1r) until the maximum RMS derivative was less than 0.001 kcal Å$^{-1}$, using Conjugate Gradient as minimization algorithm. Finally, conformations were subjected to 200 steps of unrestrained MM Conjugate Gradient energy minimization. From the produced 100 conformations, 10 structures, whose interprotonic distances best fitted NOE derived distances, were chosen for statistical analysis.

## 2. Supplementary tables

**Table S1:** Details of synthesized peptides: Purity and mass was determined via HPLC (210 nm) coupled to mass spectrometry. (F: Fluorescein isothiocyanate, Bt: Biotin, $S_5$: (*S*)-2-(4-pentenyl)alanine, $R_8$: (*R*)-2-(7-octenyl)alanine, $S_5$*: (*S*)-2-(4-pentenyl)glycine, $R_8$*: (*R*)-2-(7-octenyl)glycine, for chemical structures of non-natural amino acids see Figure S6).

| Peptide | *N*-Term. | Sequence | Purity /% | m/z calc. | m/z found |
|---|---|---|---|---|---|
| PBM | F-PEG2 | V N A K Q Y H R I L K R R Q A R A K L | 95 | 1006.0 | 1006.2 $[M+4H]^{4+}$ |
| | Ac | E A E G K I P K E R | >95 | 883.0 | 883.1 $[M+4H]^{4+}$ |
| PBM-bt | Ac | V N A K Q Y H R I L K R R Q A R A K L E A E G K I P K E R K-PEG5-Bt (2) | >95 | 1044.4 | 1044.6 $[M+4H]^{4+}$ |
| 1 | F-PEG2 | V N A K Q Y H R I L K R R Q A R A K L E A E | >95 | 1071.6 | 1071.6 $[M+3H]^{3+}$ |
| 2 | F- PEG2 | V N A K Q Y H R I L K R R Q A R A K L | 95 | 961.8 | 961.8 $[M+3H]^{3+}$ |
| | Ac | | >95 | 797.8 | 797.8 $[M+3H]^{3+}$ |
| 3 | F-PEG2 | V N A K Q Y H R I L K R R Q A R | 95 | 857.8 | 857.1 $[M+3H]^{3+}$ |
| 4 | F-PEG2 | N A K Q Y H R I L K R R Q A R A K L | 95 | 928.8 | 928.8 $[M+3H]^{3+}$ |
| 5 | F-PEG2 | K Q Y H R I L K R R Q A R A K L | 95 | 867.1 | 867.1 $[M+3H]^{3+}$ |
| 6 | F-PEG2 | Q Y H R I L K R R Q A R A K L | >95 | 824.4 | 824.4 $[M+3H]^{3+}$ |
| 2-D | F-PEG2 | V N A K Q $S_5$ H R I $S_5$ K R R Q A R A K L | >95 | 953.2 | 953.3 $[M+3H]^{3+}$ |
| | Ac | | >95 | 789.2 | 789.2 $[M+3H]^{3+}$ |
| 2-C | F-PEG2 | V N A K Q Y $R_8$ R I L K R R $S_5$ A R A K L | >95 | 970.9 | 971.1 $[M+3H]^{3+}$ |
| | Ac | | >95 | 806.8 | 807.0 $[M+3H]^{3+}$ |
| 2-B | F-PEG2 | V N A K Q Y H R I $S_5$ K R R $S_5$ A R A K L | >95 | 964.9 | 965.1 $[M+3H]^{3+}$ |
| 2-A | F-PEG2 | V N A K Q Y H R I $R_8$ K R R Q A R $S_5$ K L | >95(1) | 997.9 | 997.9 $[M+3H]^{3+}$ |
| 2-D$^N$ | F-PEG2 | V N A K Q $S_5^*$ H R I $S_5$ K R R Q A R A K L | >95 | 948.6 | 948.6 $[M+3H]^{3+}$ |
| | Ac | | >95 | 784.5 | 784.6 $[M+3H]^{3+}$ |
| 2-D$^C$ | F-PEG2 | V N A K Q $S_5$ H R I $S_5^*$ K R R Q A R A K L | >95 | 948.5 | 948.7 $[M+3H]^{3+}$ |
| 5-D | F-PEG2 | K Q $S_5$ H R I $S_5$ K R R Q A R A K L | >95 | 858.5 | 858.5 $[M+3H]^{3+}$ |
| 5-C | F-PEG2 | K Q Y $R_8$ R I L K R R $S_5$ A R A K L | >95 | 876.2 | 876.2 $[M+3H]^{3+}$ |
| 5-B | F-PEG2 | K Q Y H R I $S_5$ K R R $S_5$ A R A K L | >95 | 870.1 | 870.4 $[M+3H]^{3+}$ |
| 5-A | F-PEG2 | K Q Y H R I $R_8$ K R R Q A R $S_5$ K L | >95(1) | 903.2 | 903.1 $[M+3H]^{3+}$ |
| 5-D$^N$ | F-PEG2 | K Q $S_5^*$ H R I $S_5$ K R R Q A R A K L | >95 | 853.5 | 853.7 $[M+3H]^{3+}$ |
| 2-C$^N$ | F-PEG2 | V N A K Q Y $R_8^*$ R I L K R R $S_5$ A R A K L | >95 | 966.2 | 966.1 $[M+3H]^{3+}$ |
| 2-C$^C$ | F-PEG2 | V N A K Q Y $R_8$ R I L K R R $S_5^*$ A R A K L | >95 | 966.2 | 966.3 $[M+3H]^{3+}$ |

(1) HPLC chromatogram indicates two isomers.
(2) Attached via sidechain.

**Table S2:** Crystallographic data and refinement statistics of NF-YB/C in complex with **PBM** (PDB ID: 6qmp), **2-C** (PDB ID: 6qms) and **2-D^N^** (PDB ID: 6qmq). Values in parenthesis correspond to the highest resolution shell.

| | NF-YB/NF-YC/**PBM** | NF-YB/NF-YC/**2-C** | NF-YB/NF-YC/**2-D^N^** |
|---|---|---|---|
| **PDB ID** | 6qmp | 6qms | 6qmq |
| **Data collection** | | | |
| Space group | $P3_221$ | $P2_12_12_1$ | $P2_12_12_1$ |
| Cell dimensions | | | |
| a, b, c (Å) | 52.9, 52.9, 134,1 | 48.9, 49.8, 74.5 | 45.0, 52.2, 73.0 |
| α, β, γ (°) | 90, 90, 120 | 90, 90, 90 | 90, 90, 90 |
| Wavelength (Å) | 0.979 | 0.978 | 0.978 |
| Resolution limits (Å) | 50 - 2.0 (2.1 - 2.0) | 50 - 1.8 (1.9 - 1.8) | 50 - 2.5 (2.6 - 2.5) |
| No. of unique relections | 15380 (2046) | 17408 (2544) | 6325 (682) |
| Completeness (%) | 99.8 (100) | 99.4 (99.0) | 99.6 (100) |
| Multiplicity | 18.88 (18.86) | 12.38 (12.79) | 12.18 (12.93) |
| I / σI | 21.85 (2.81) | 20.16 (2.15) | 11.07 (2.18) |
| CC1/2 | 99.9 (84.6) | 99.9 (89.5) | 99.7 (66.5) |
| Robs | 7.2 (121.1) | 6.2 (190.9) | 15.4 (139.4) |
| **Refinement** | | | |
| Resolution limits (Å) | 50 - 2.0 (2.1 - 2.0) | 50 - 1.8 (1.9 - 1.8) | 50 - 2.5 (2.6 - 2.5) |
| $R_{work}$ / $R_{free}$ | 0.2129 / 0.2731 | 0.2036 / 0.2339 | 0.1884 / 0.2728 |
| R.m.s. deviations | | | |
| Bond lengths (Å) | 0.007 | 0.006 | 0.009 |
| Bond angles (°) | 0.861 | 0.829 | 1.169 |
| B-factor $Å^2$ | 48.62 | 36.09 | 55.57 |
| No. atoms | | | |
| Protein | 1336 | 1334 | 1293 |
| Peptide | 210 | 156 | 150 |
| Ligand/ion | 11 | 0 | 1 |
| Water | 36 | 98 | 26 |
| Ramachandran | | | |
| Favored Regions (%) | 96.2 | 98.8 | 95.5 |
| Allowed Regions (%) | 3.8 | 1.2 | 4.5 |
| Outliers (%) | 0 | 0 | 0 |

**Table S3**: Isothermal titration calorimetry data for the peptides **2**, **2-C**, **2-D** and **2-D$^N$** binding to NF-YB/C.

| Peptide | $K_D$/ µM | N | $\Delta H$ / kcal mol$^{-1}$ | $\Delta S$ / cal mol$^{-1}$ K$^{-1}$ | -T$\Delta S$ / kcal mol$^{-1}$ | $\Delta G$ / kcal mol$^{-1}$ |
|---|---|---|---|---|---|---|
| **2** | 1.37 ±0.03 | 0.97 ±0.01 | -21.0 ±0.4 | -44 ±1 | 13.0 ±0.4 | -7.99 ±0.02 |
| **2-C** | 1.08 ±0.06 | 1.03 ±0.06 | -13.0 ±0.1 | -16.4 ±0.5 | 4.9 ±0.2 | -8.15 ±0.04 |
| **2-D** | 5.7 ±0.5 | 1.26 ±0.04 | -12.3 ±0.1 | -17.1±0.5 | 5.1 ±0.1 | -7.15 ±0.04 |
| **2-D$^N$** | 0.37 ±0.01 | 0.76 ±0.08 | -24.8 ±0.3 | -53.8 ±0.8 | 16.0 ±0.3 | -8.78 ±0.03 |

**Table S4:** Secondary structure distribution obtained from circular dichroism measurements. Calculated with CDNN/PEPFIT.

| Peptide | Helix | β-Sheet (antiparalell) | β-Sheet (paralell) | β-Turn | Random coil | Total sum |
|---|---|---|---|---|---|---|
| **2** | 13 | 57 | 0 | 3.6 | 59 | 133 |
| **2-C** | 81 | 2 | 0.1 | 5.6 | 19 | 108 |
| **2-D** | 47 | 9.5 | 0 | 8.1 | 35 | 100 |
| **2-D$^N$** | 47 | 11 | 0 | 7.6 | 35 | 101 |

**Table S5:** Table of intramolecular NOEs diagnostic of folded structures from **2-D** and **2-D^N^** bound to NF-YB/C. (*NOEs not observed in solution). [#]NOEs detected from protons of α-methyl group (Hβ(Me)/position 272) in peptide **2-D** and the corresponding residue of **2-D^N^** (Hα).

| NOE | Residue 1 | Atom 2 | Residue 2 | Atom 2 | 2D | 2D$^N$ |
|---|---|---|---|---|---|---|
| 1 | N268 | Hα | K270 | HN | + | + |
| 2 | N268 | Hα | Q271 | HN | + | |
| 3 | N268 | CONH2 | Q271 | Hγ | + | + |
| 4 | A269 | Hα | Q271 | HN | + | |
| 5 | A269 | Hα | X272 | HN | + | + |
| 6 | Q271 | Hα | R274 | HN | + | +* |
| 7 | X272 | HN | R274 | HN | + | +* |
| 8 | X272 | Hβ(Me) | R274 | HN | + | |
| 9 | X272 | Hβ(Me)/Hα[#] | I275 | HN | + | + |
| 10 | X272 | Hβ(Me)/Hα[#] | I275 | Hβ | + | +* |
| 11 | X272 | Hβ(Me)/Hα[#] | I275 | Hδ | + | + |
| 12 | H273 | Hα | X276 | HN | | +* |
| 13 | H273 | Hα | K277 | HN | + | + |
| 14 | R274 | Hα | X276 | HN | + | |
| 15 | R274 | HN | X276 | HN | + | |
| 16 | I275 | Hα | K277 | HN | + | |
| 17 | I275 | Hα | R278 | HN | + | + |
| 18 | I275 | Hα | R278 | Hβ | + | |
| 19 | I275 | Hα | R279 | HN | +* | + |
| 20 | X276 | HN | R278 | HN | + | |
| 21 | X276 | Hβ(Me) | R279 | HN | + | + |
| 22 | K277 | Hα | R279 | HN | + | + |
| 23 | K277 | Hα | Q280 | HN | + | + |
| 24 | K277 | Hα | A281 | HN | | + |
| 25 | Q280 | Hα | K284 | Hα | + | +* |

## 3. Supplementary figures

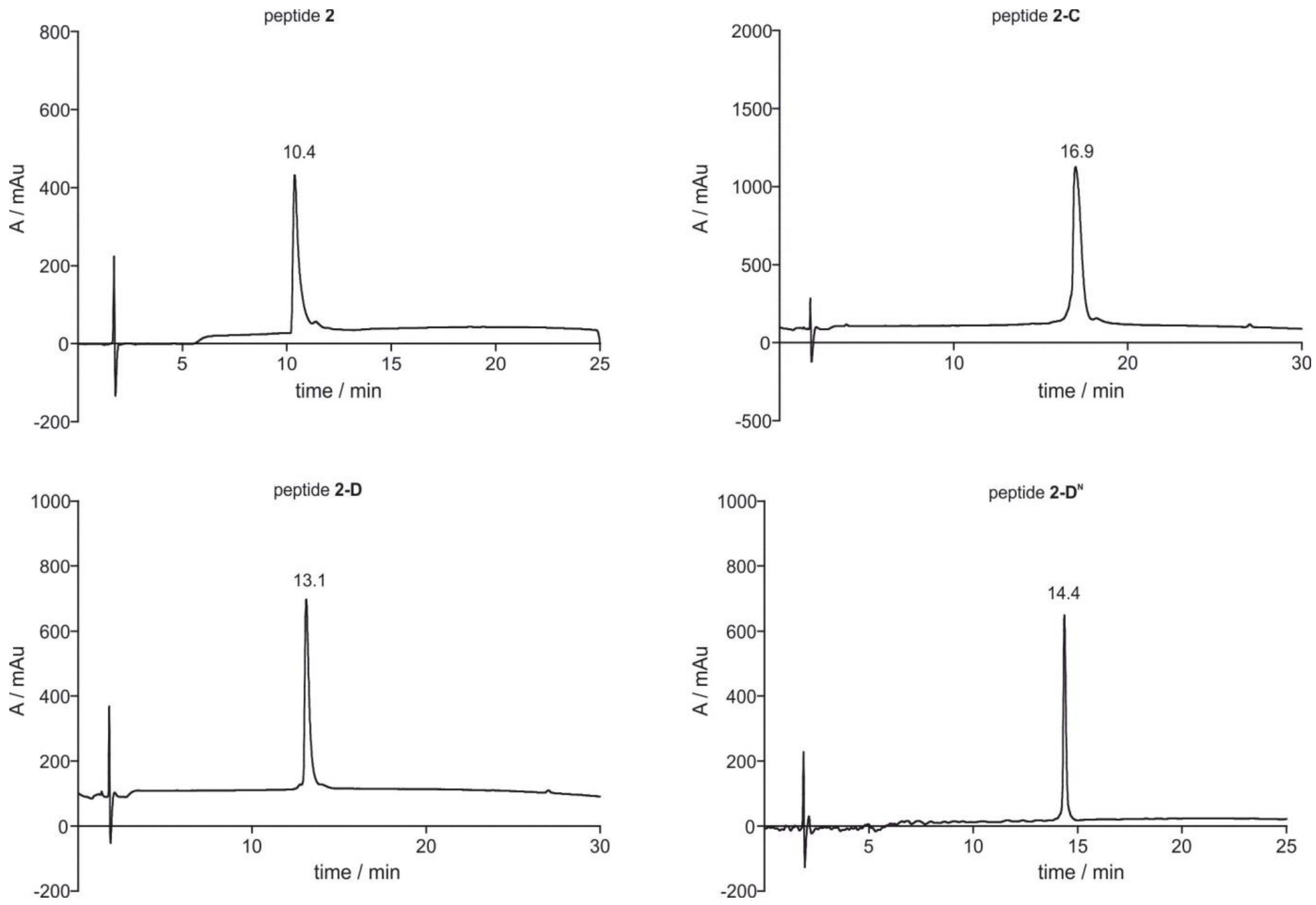


**Figure S1:** HPLC Chromatogram (λ = 210 nm) of *N*-terminally acetylated peptides **2**, **2-C**, **2-D**, **2-D$^{N}$** as used in ITC, X-ray, NMR and competition assays.

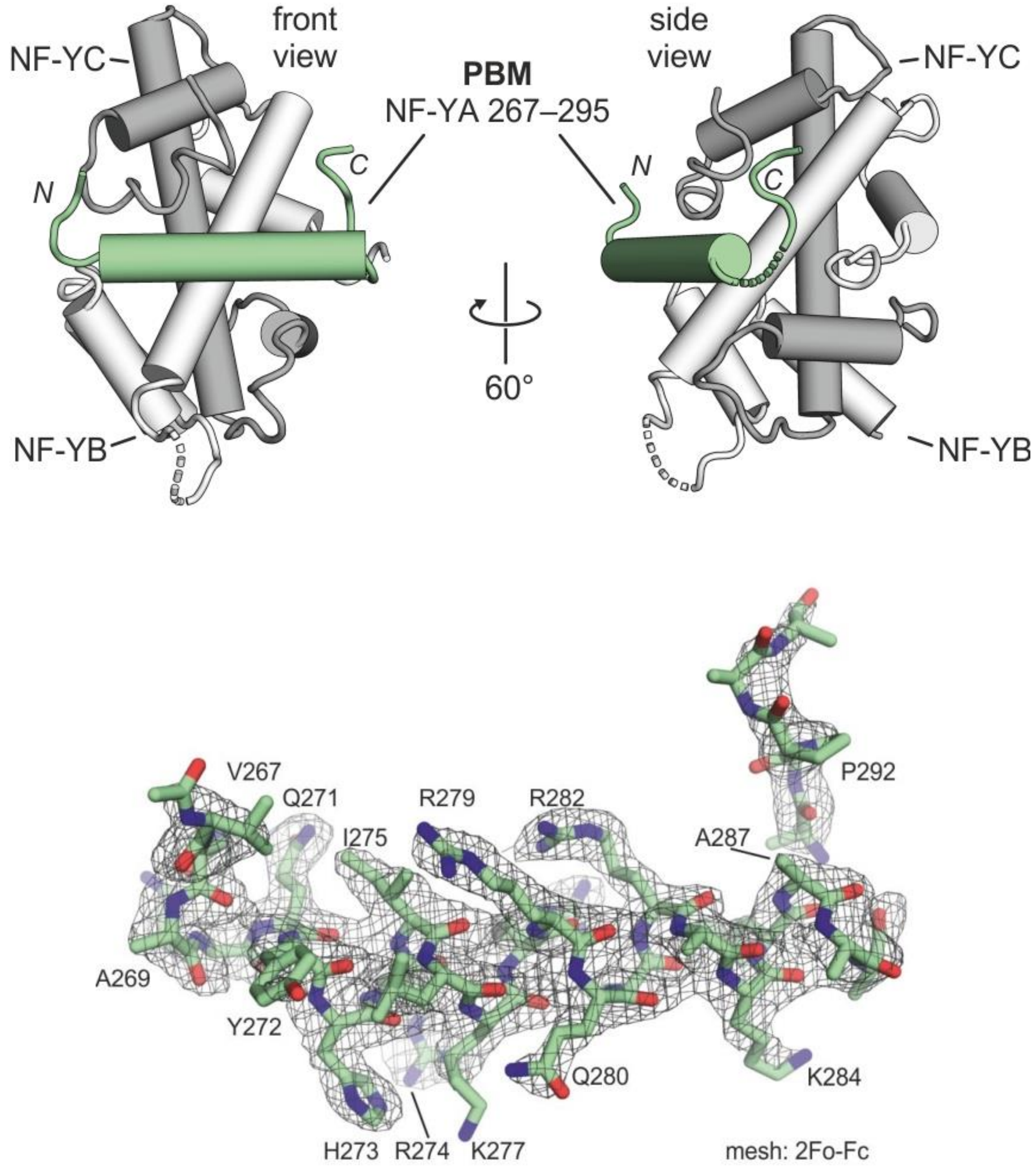


**Figure S2:** Top: Crystal structure (PDB ID: 6qmp, Table S2) of **PBM** (V267–R295, green) in complex with the NF-YB/C dimer (grey) in cartoon representation. Bottom: Crystal structure of **PBM** (NF-YA V267 – R295) originating from the complex with NF-YB/C (PDB ID: 6qmp). Electron density is shown as mesh (2Fo-Fc, level 1σ).

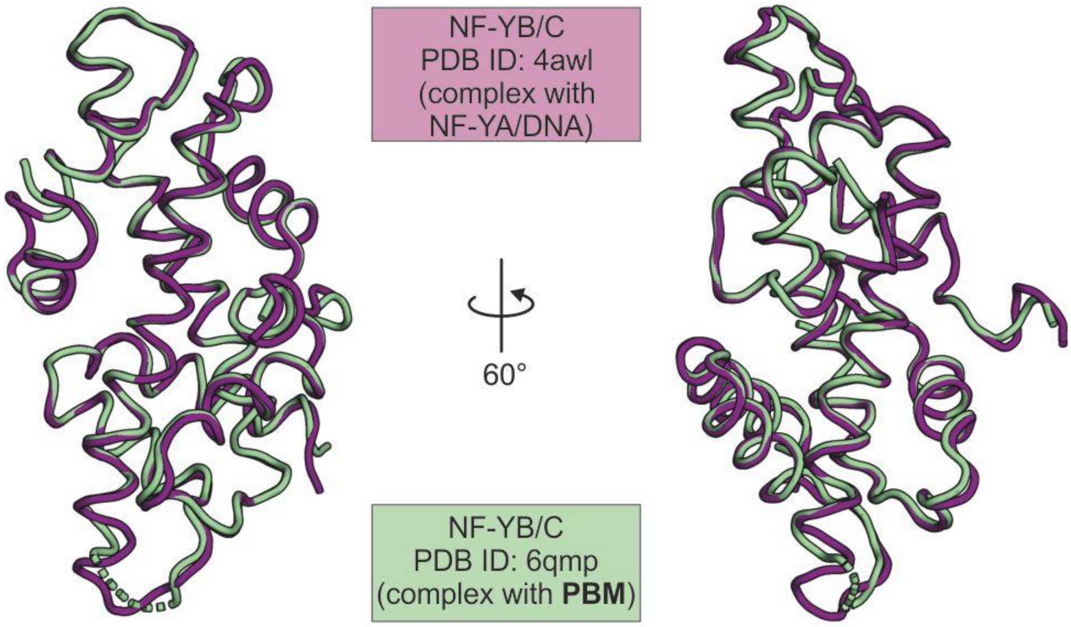


**Figure S3:**. Superimposition of NF-YB/C dimer originating from the complex with **PBM** (PDB ID: 6qmp, green) and from the complex with NF-YA and DNA (PDB ID: 4awl, purple).[3] RMSD: 0.85 Å. Number of aligned residues: 169.

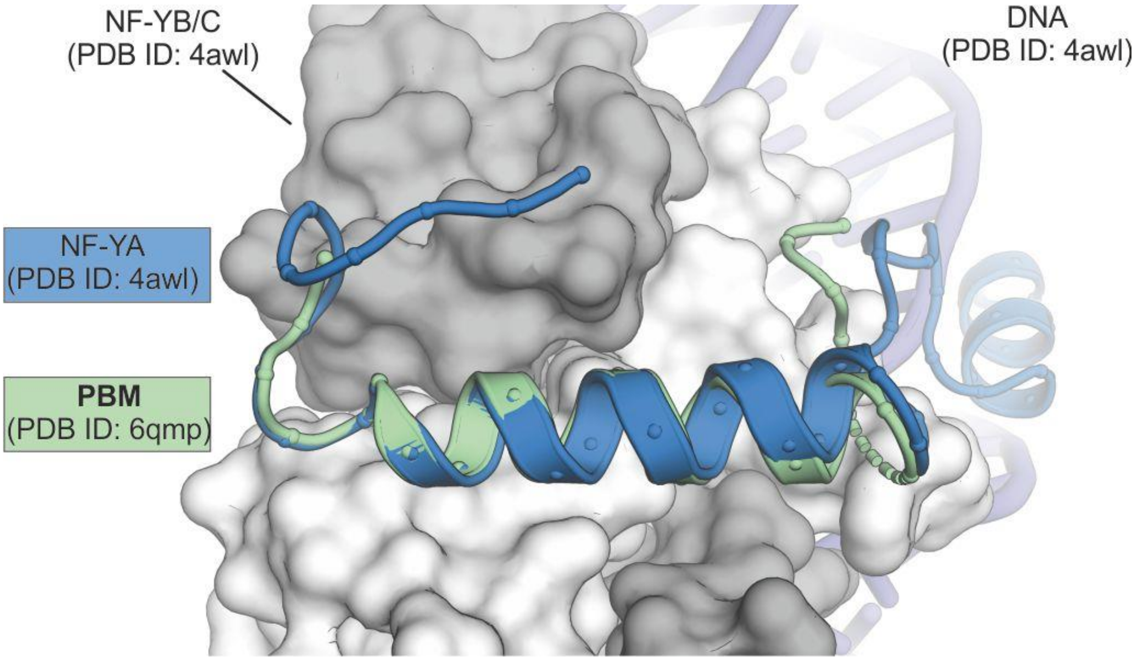


**Figure S4:** Superimposition of **PBM** (green) in complex with NF-YB/C (PDB ID: 6qmp) and of NF-YA (blue) in complex with NF-YB/C and DNA (PDB ID: 4awl). RMSD over all 27 residues: 1.56 Å, RMSD for residues V267 – E288: 0.52 Å.

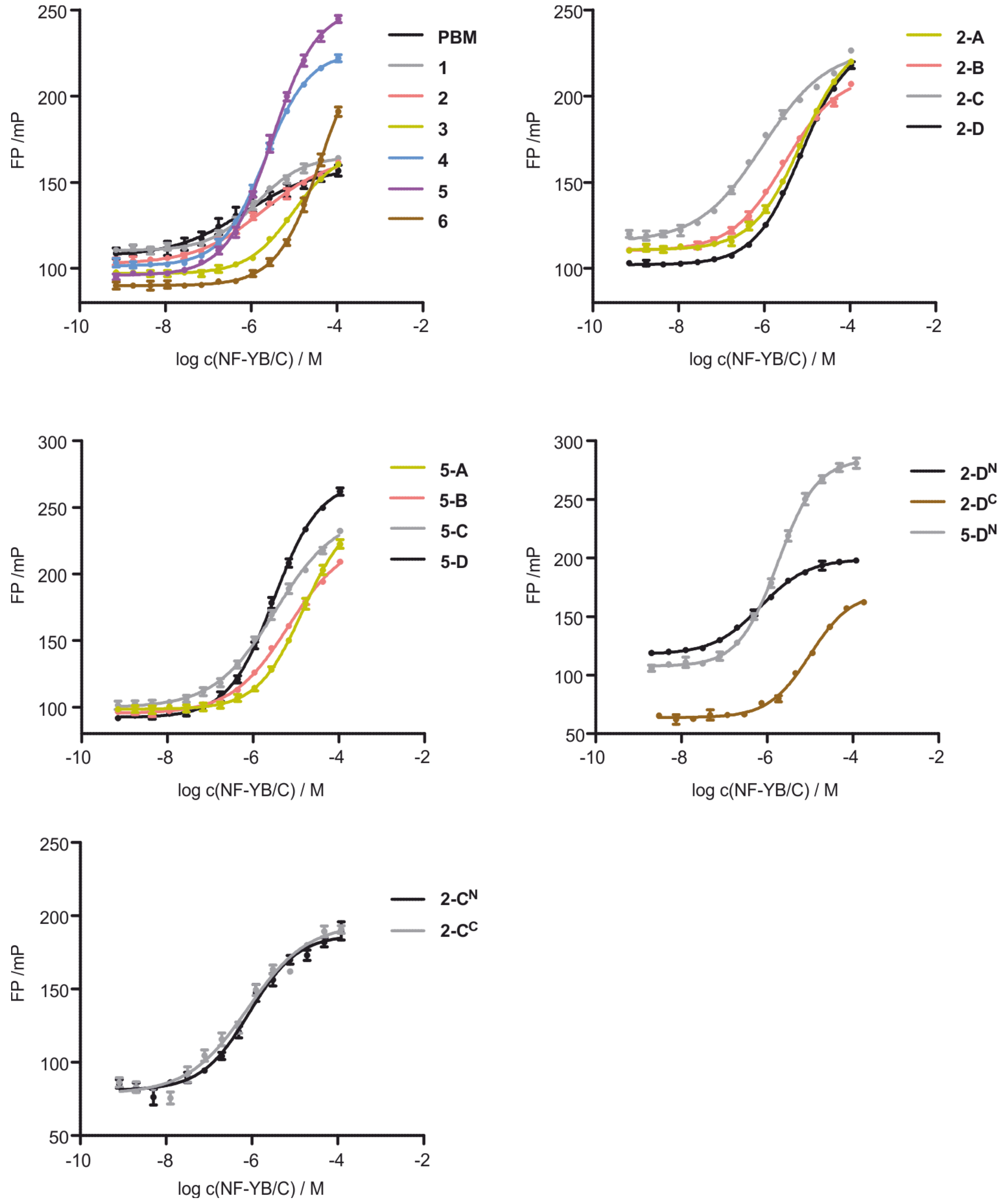


**Figure S5:** Fluorescence polarisation measurements of FITC-labelled peptides binding to NF-YB/C. Measurements were performed as triplicates (c(peptide) = 10 nM, c(NF-YB/C) = $7.1\cdot10^{-10} – 1.8\cdot10^{-4}$ M).

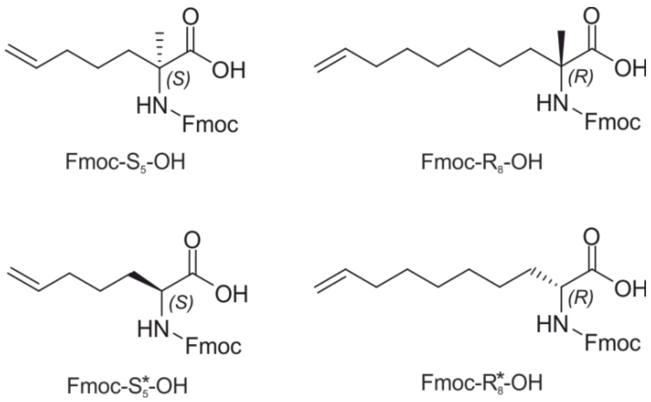


**Figure S6:** Fmoc-protected, non-natural amino acids as used in solid-phase peptide synthesis (Fmoc-$S_5$-OH: Fmoc-(*S*)-2-(4-pentenyl)-alanine; Fmoc-$R_8$-OH: Fmoc-(*R*)-2-(7-octenyl)alanine; Fmoc-$S_5$*-OH: Fmoc-(*S*)-2-(4-pentenyl)glycine); $R_8$*: (*R*)-2-(7-octenyl)glycine).

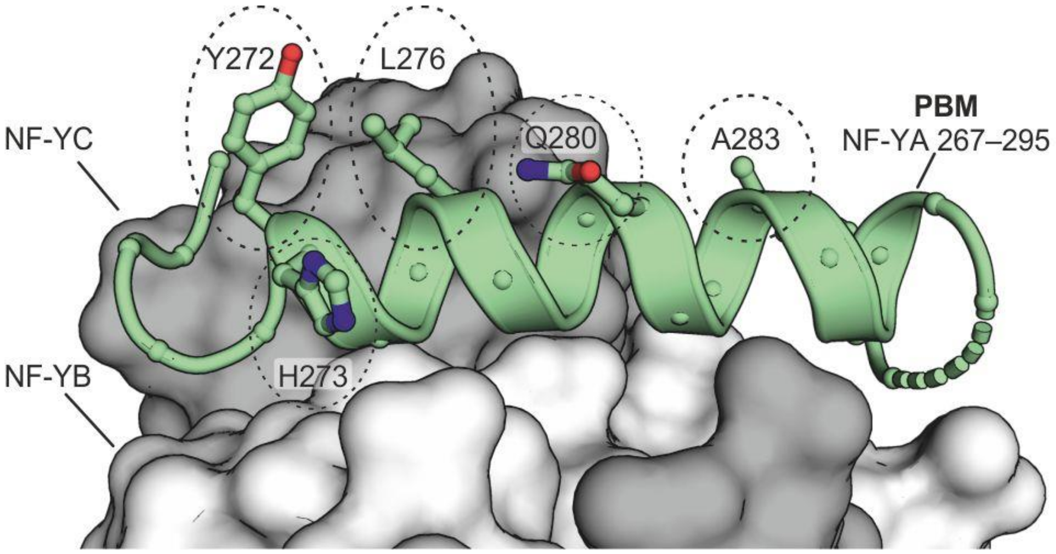


**Figure S7**: Crystal structure of **PBM** (NF-YA 267–295) in complex with NF-YB/C (PDB ID: 6qmp). Highlighted residues were chosen to be substituted by non-natural amino acids for peptide macrocyclization.

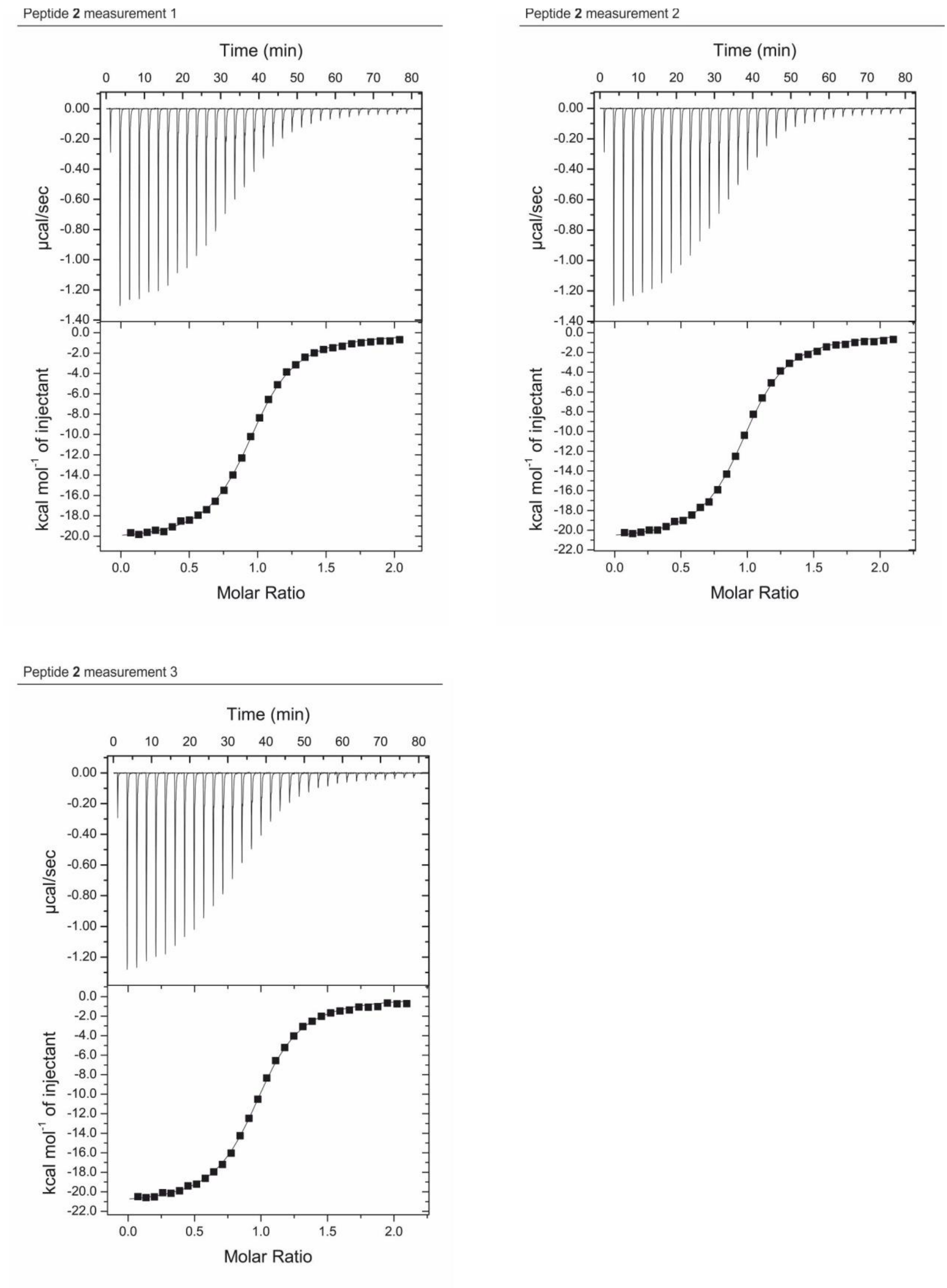


**Figure S8:** ITC measurements of peptide **2**. Measurements were performed as triplicates (c(peptide) = 50 µM, c(NF-YB/C) = 500 µM).

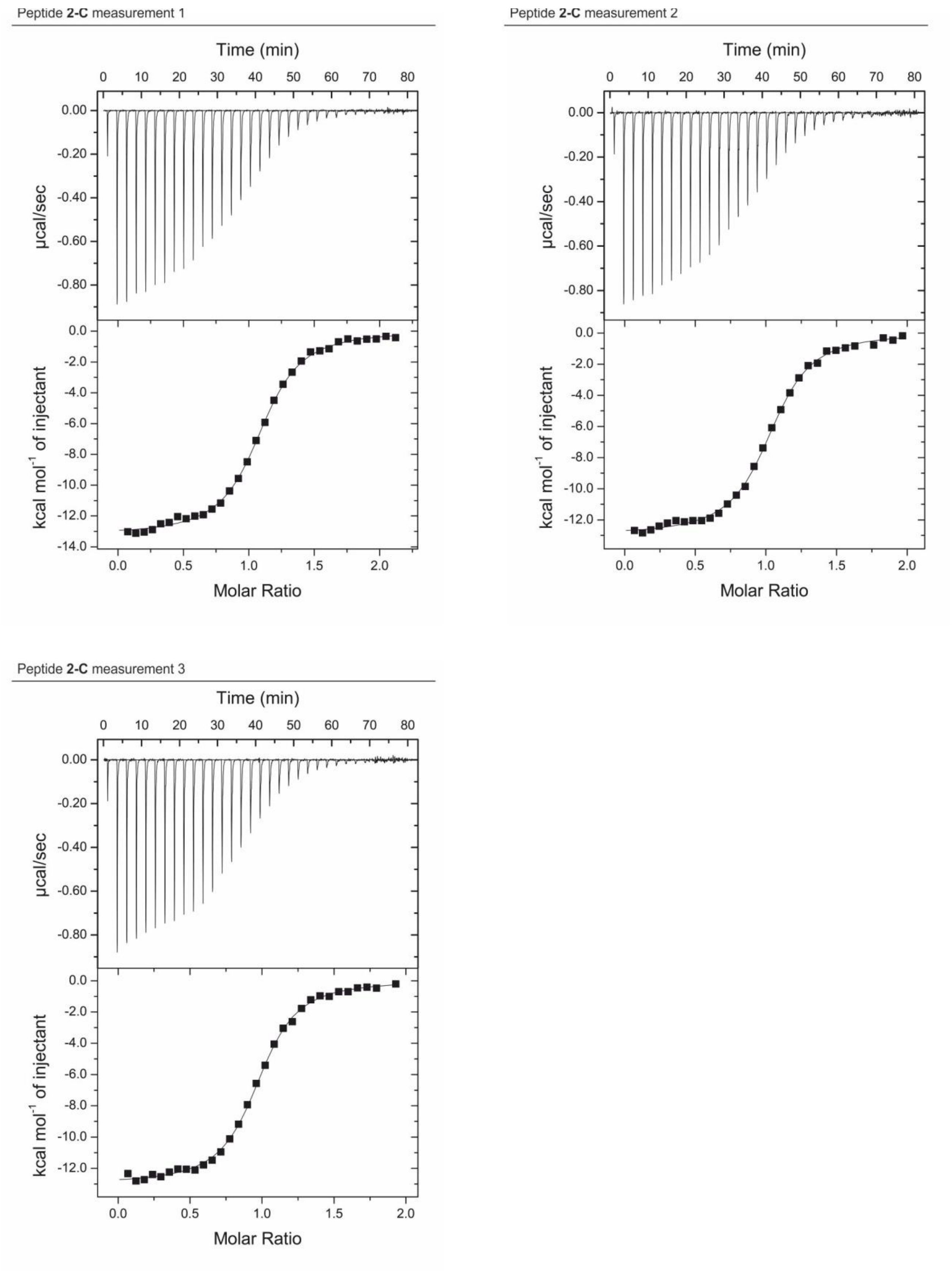


**Figure S9:** ITC measurements of peptide **2-C**. Measurements were performed as triplicates (c(peptide) = 50 µM, c(NF-YB/C) = 500 µM).

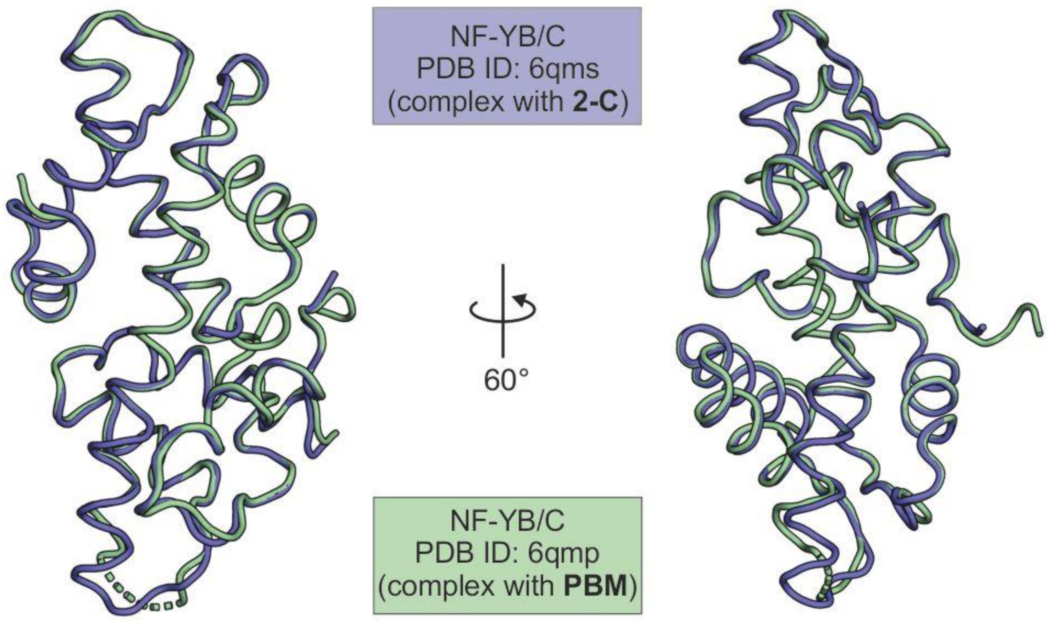


**Figure S10**: Superimposition of NF-YB/C dimer originating from the complex with **PBM** (PDB ID: 6qmp, green) and from the complex with **2-C** (PDB ID: 6qms, blue). RMSD: 0.74 Å (number of aligned residues: 166).

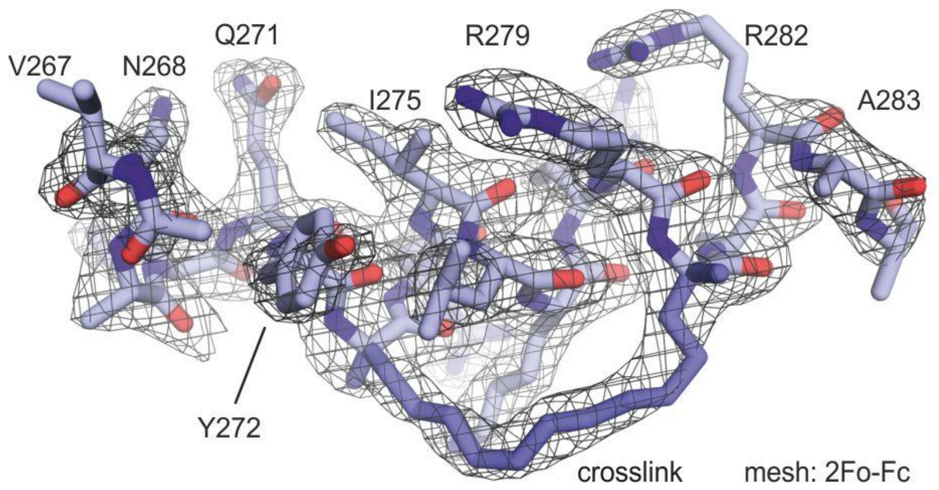


**Figure S11:** Crystal structure of **2-C** originating from a complex with NF-YB/C (PDB ID: 6qms). Electron density is shown as mesh (2Fo-Fc, level 1σ).

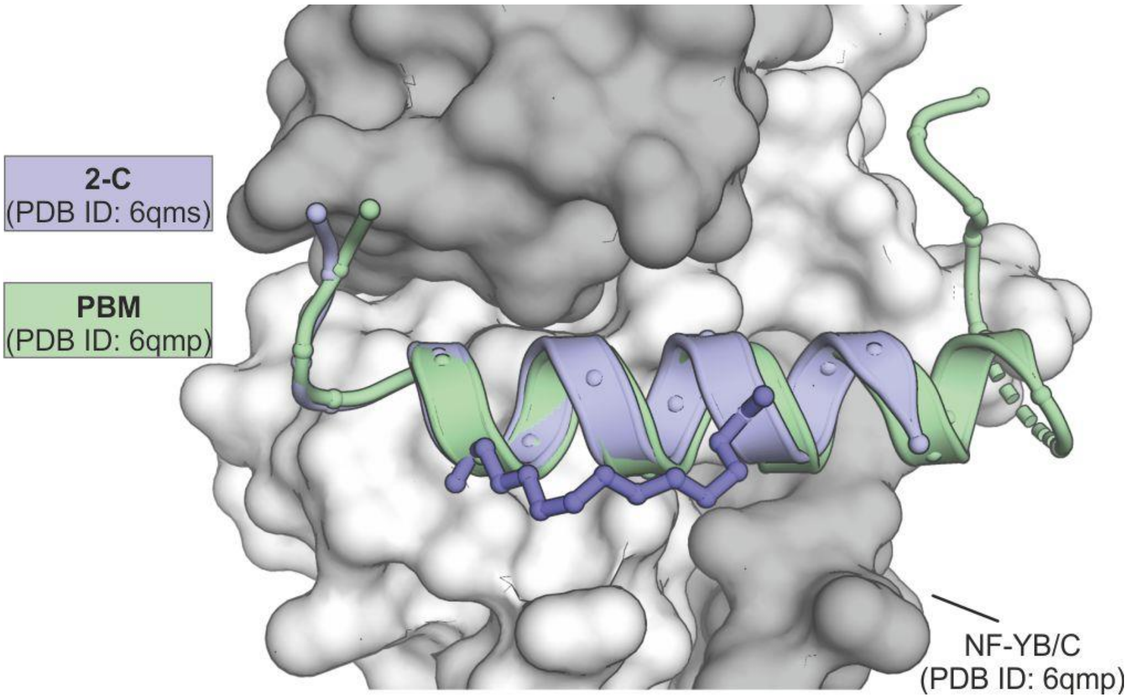


**Figure S12**: Superimposition of **2-C** (blue, PDB ID 6qms) and **PBM** (green, PDB ID: 6qmp) in complex with NF-YB/C. RMSD of peptide alignment: 0.59 Å (number of aligned residues: 18).

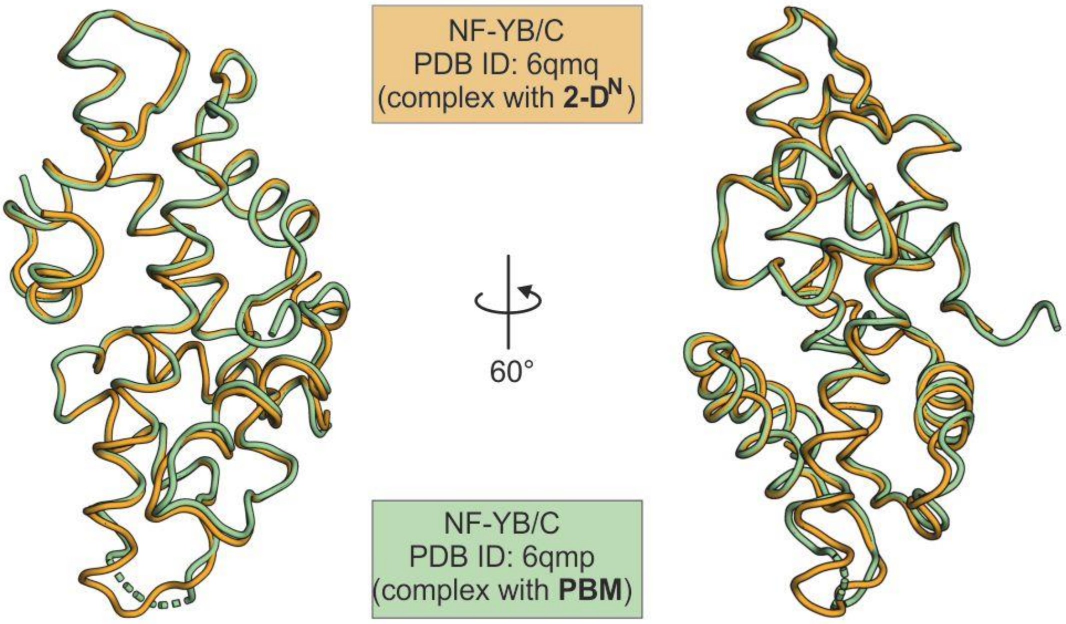


**Figure S13:** Superimposition of NF-YB/C dimer originating from the complex with **PBM** (PDB ID: 6qmp, green) and from the complex with **2-D$^{\mathbf{N}}$** (PDB ID: 6qmq, orange). RMSD: 0.91 Å (number of aligned residues: 168).

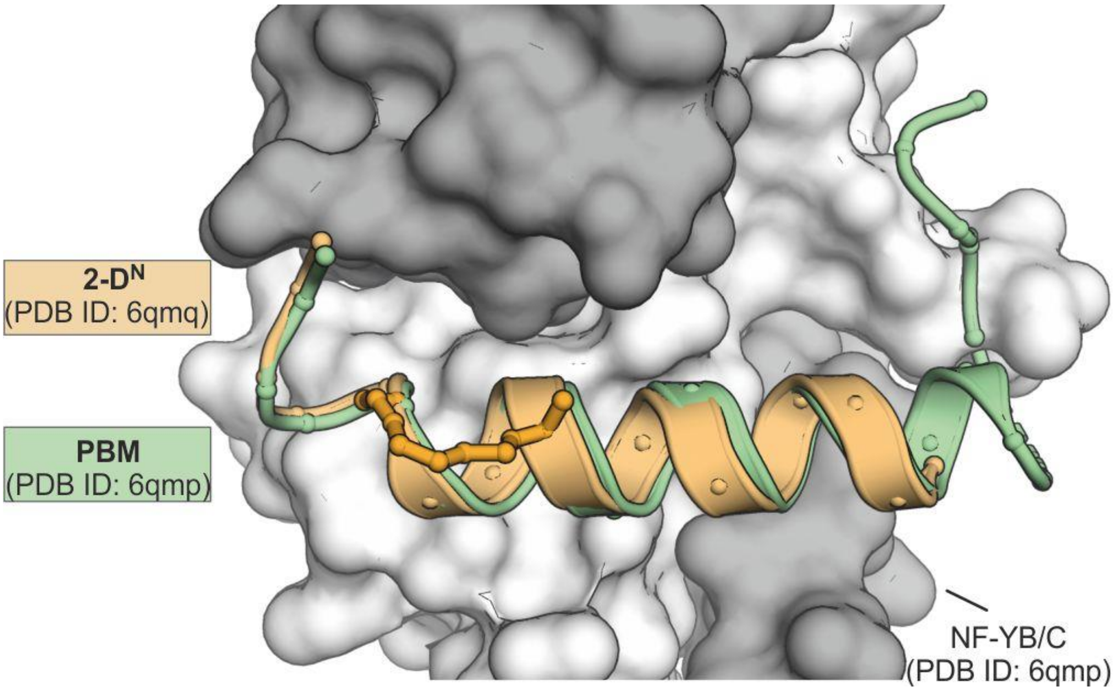


**Figure S14**: Superimposition of **2-D$^{N}$** (orange, PDB ID: 6qmq) and **PBM** (green, PDB ID: 6qmp) in complex with NF-YB/C. RMSD of peptide alignment: 0.37 Å (number of aligned residues: 19).

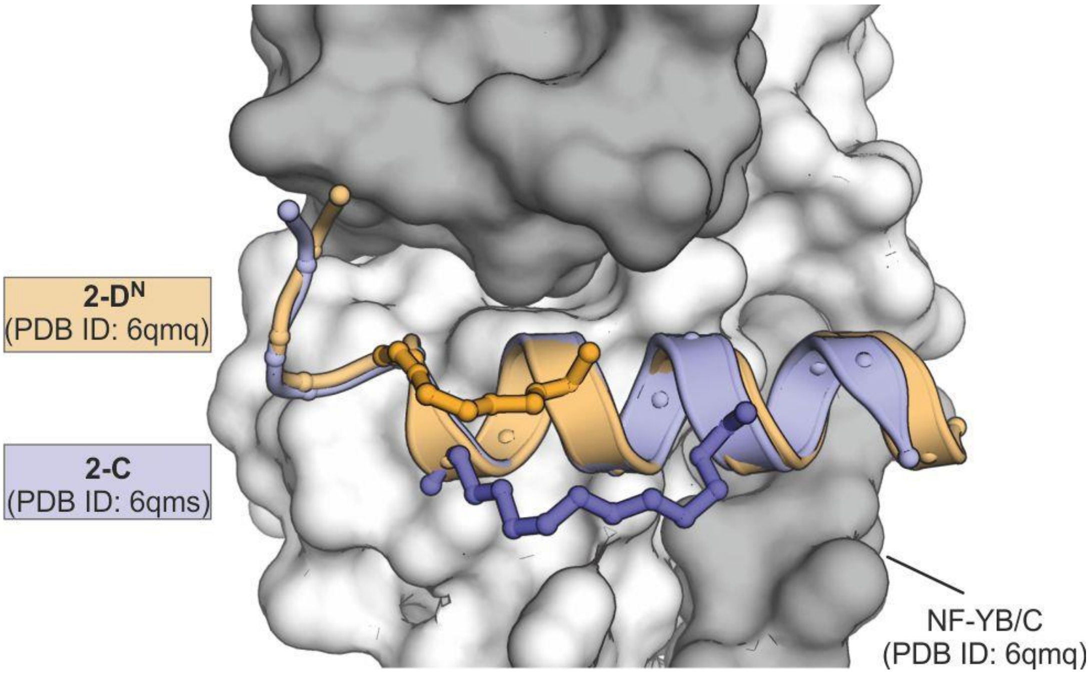


**Figure S15**: Superimposition of **2-D$^{N}$** (orange, PDB ID: 6qmq) and **2-C** (blue, PDB ID: 6qms) in complex with NF-YB/C. RMSD of peptide alignment: 0.59 Å (umber of aligned residues: 18).

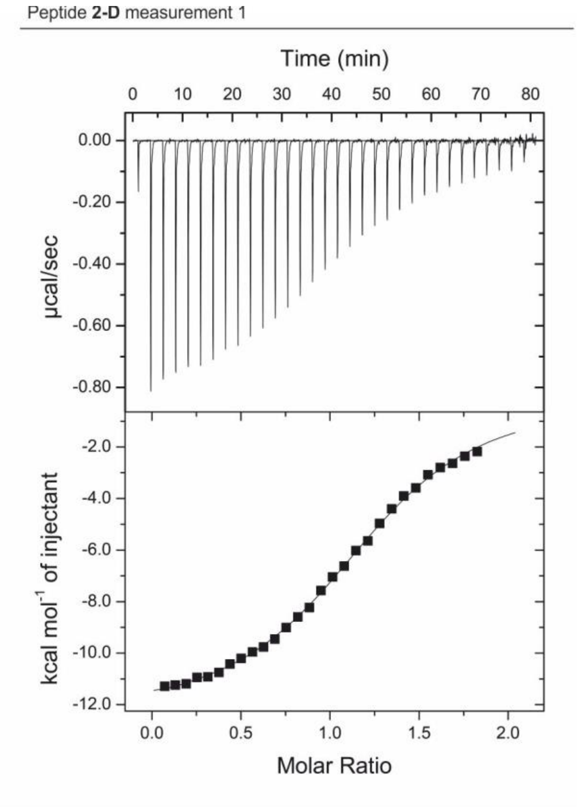


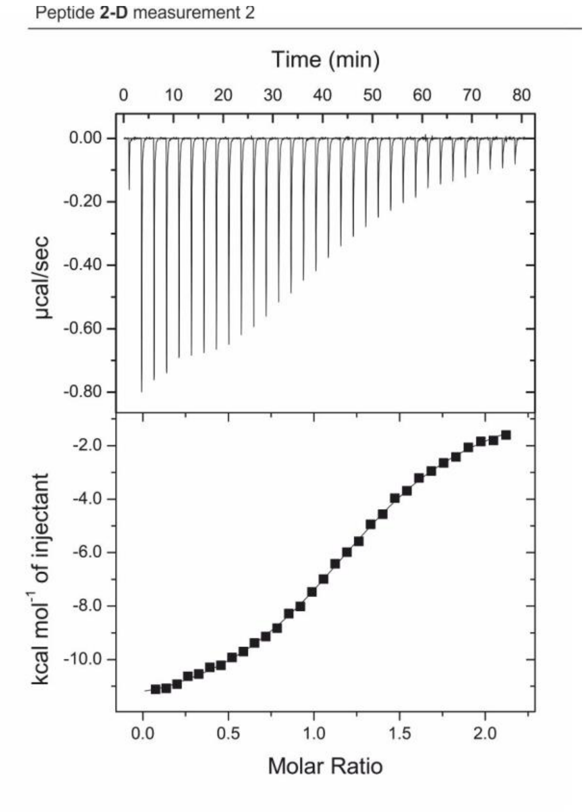


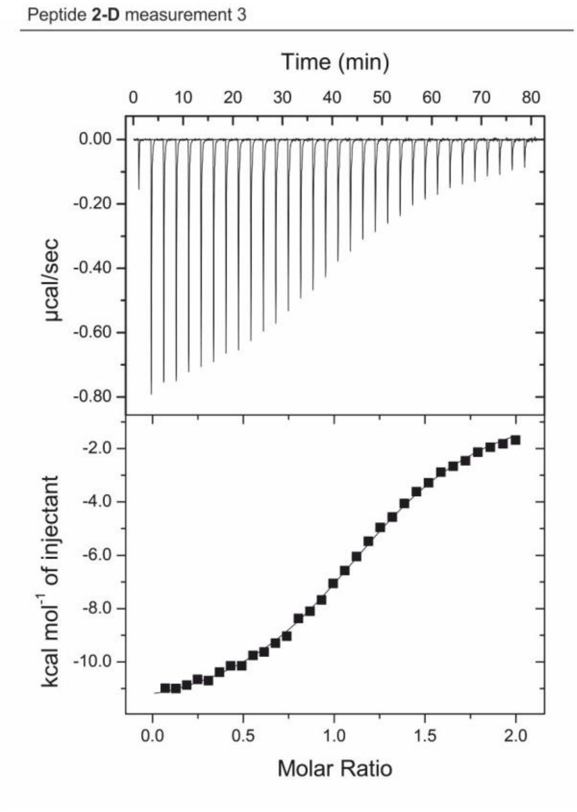


**Figure S16:** ITC measurements of peptide **2-D**. Measurements were performed as triplicates (c(peptide) = 50 µM, c(NF-YB/C) = 500 µM).

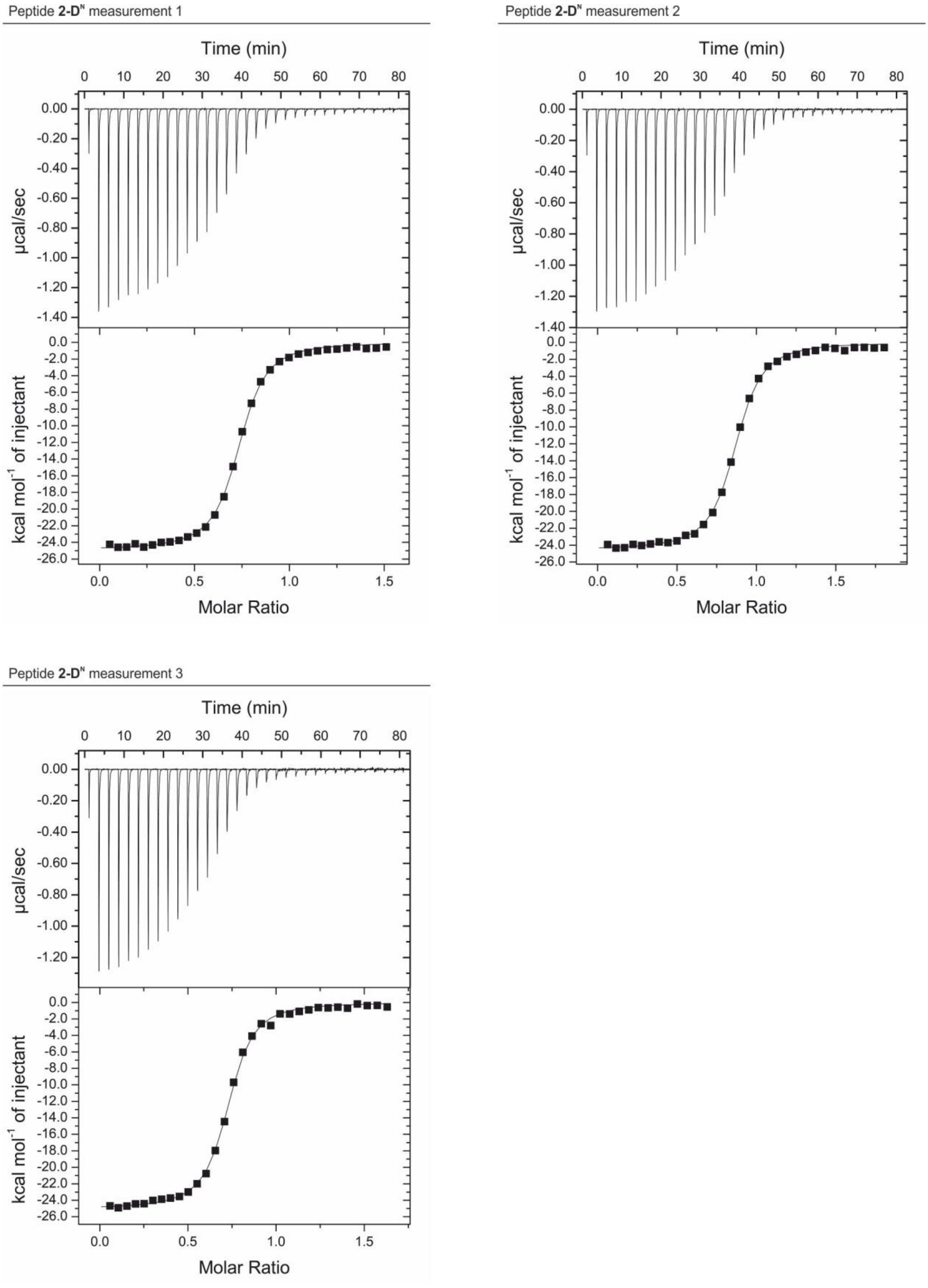


**Figure S17:** ITC measurements of peptide **2-D$^{\mathbf{N}}$**. Measurements were performed as triplicates (c(peptide) = 50 µM, c(NF-YB/C) = 500 µM).

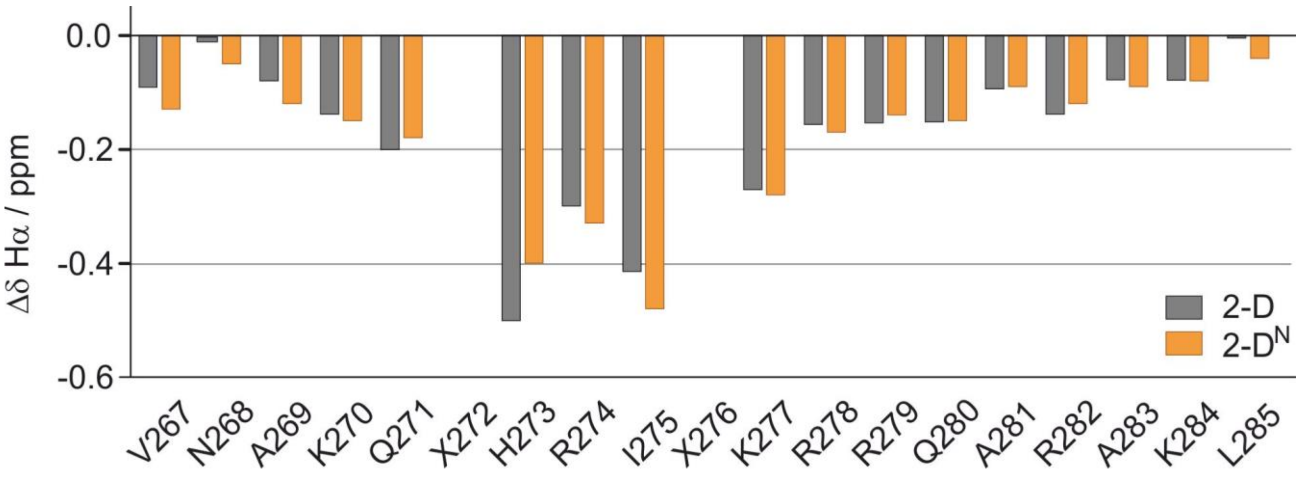


**Figure S18:** Chemical shift deviations from random coil reference of Hα for **2-D** (grey) and **2-D$^{N}$** (orange).

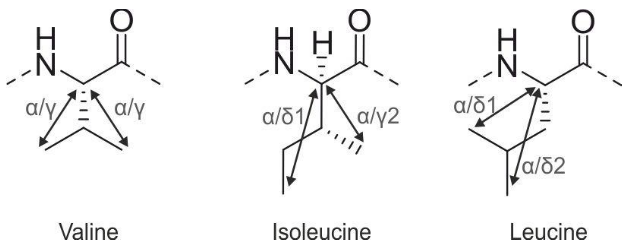


**Figure S19:** 2-D $^{1}$H-$^{1}$H coupling (TOCSY) of residues V267, I275, L285 in manuscript Figure 4A.

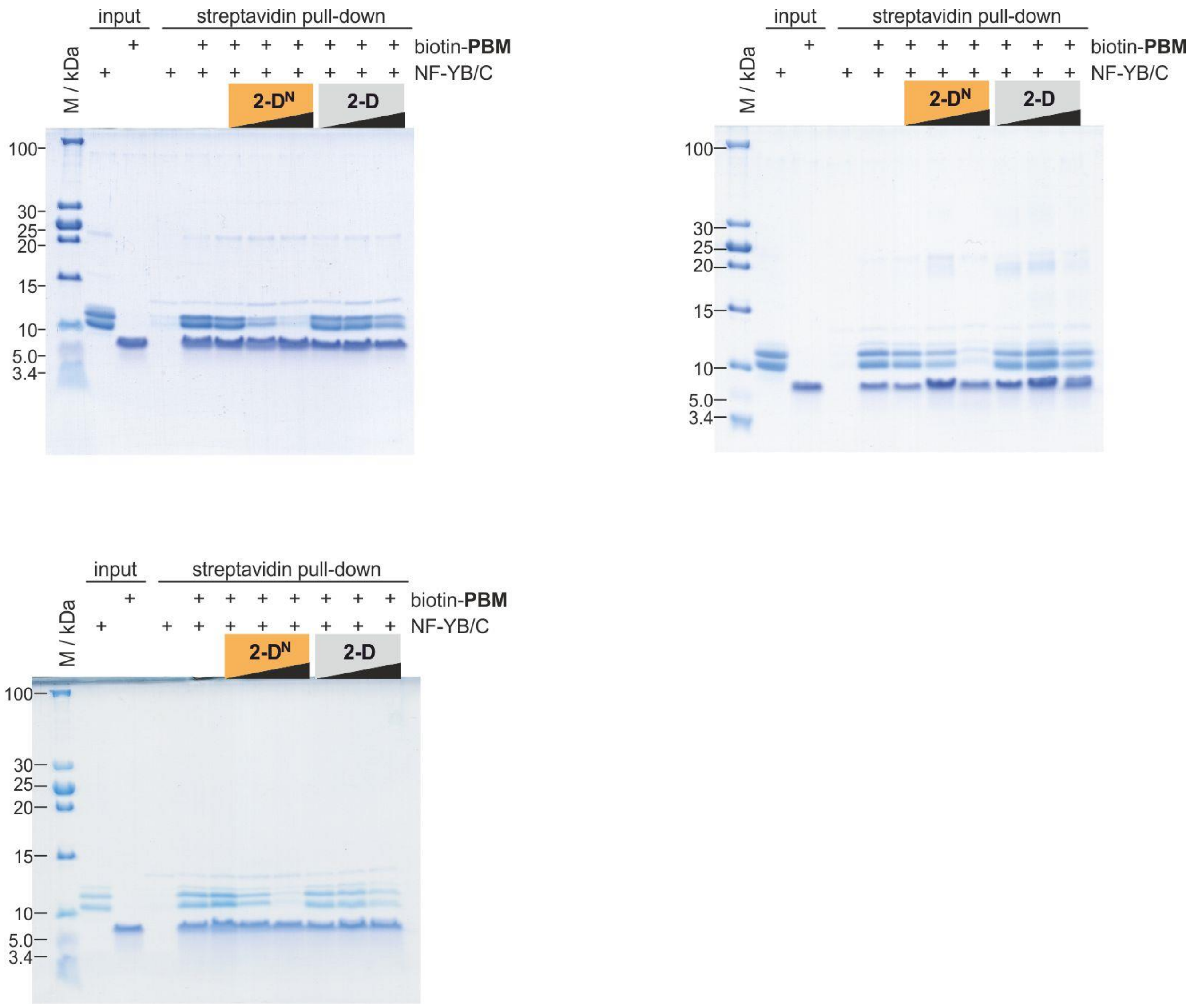


**Figure S20:** Pull-down experiment: Immobilised **PBM** on Streptavidin beads binding to NF-YB/C (80 µM) in absence and presence of peptide ($c$ = 16 µM, 80 µM, 400 µM). Assay was performed as triplicate.

## 4. References


[1] Y. W. Kim, T. N. Grossmann, G. L. Verdine, *Nat. Protoc.* **2011**, *6*, 761-771.

[2] M. Z. Li, S. J. Elledge, *Methods Mol. Biol.* **2012**, *852*, 51-59.

[3] M. Nardini, N. Gnesutta, G. Donati, R. Gatta, C. Forni, A. Fossati, C. Vonrhein, D. Moras, C. Romier, M. Bolognesi, R. Mantovani, *Cell* **2013**, *152*, 132-143.

[4] W. Kabsch, *Acta Crystallogr. D Biol. Crystallogr.* **2010**, *66*, 125-132.

[5] A. J. McCoy, *Acta Crystallogr. D Biol. Crystallogr.* **2007**, *63*, 32-41.

[6] P. Emsley, B. Lohkamp, W. G. Scott, K. Cowtan, *Acta Crystallogr. D Biol. Crystallogr.* **2010**, *66*, 486-501.

[7] P. D. Adams, P. V. Afonine, G. Bunkóczi, V. B. Chen, I. W. Davis, N. Echols, J. J. Headd, L.-W. Hung, G. J. Kapral, R. W. Grosse-Kunstleve, A. J. McCoy, N. W. Moriarty, R. Oeffner, R. J. Read, D. C. Richardson, J. S. Richardson, T. C. Terwilliger, P. H. Zwart, *Acta Crystallogr. D Biol. Crystallogr.* **2010**, *66*, 213-221.

[8] a) B. C. Poschner, J. Reed, D. Langosch, M. W. Hofmann, *Anal. Biochem.* **2007**, *363*, 306-308; b) G. Bohm, R. Muhr, R. Jaenicke, *Protein Eng.* **1992**, *5*, 191-195.

[9] T. L. Hwang, A. J. Shaka, *J. Magn. Reson. A* **1995**, *112*, 275-279.

[10] a) U. Piantini, O. W. Sorensen, R. R. Ernst, *J. Am. Chem. Soc.* **1982**, *104*, 6800-6801; b) D. Marion, K. Wüthrich, *Biochem. Biophys. Res. Commun.* **1983**, *113*, 967-974.

[11] L. Braunschweiler, R. R. Ernst, *J. Magn. Reson. (1969)* **1983**, *53*, 521-528.

[12] J. Jeener, B. H. Meier, P. Bachmann, R. R. Ernst, *J. Chem. Phys.* **1979**, *71*, 4546-4553.

[13] D. J. States, R. A. Haberkorn, D. J. Ruben, *J. Magn. Reson. (1969)* **1982**, *48*, 286-292.

[14] C. Bartels, T. H. Xia, M. Billeter, P. Guntert, K. Wuthrich, *J. Biomol. NMR* **1995**, *6*, 1-10.

[15] K. Wüthrich, *NMR of proteins and nucleic acids*, John Wiley & Sons, New York, **1986**.

[16] P. Guntert, C. Mumenthaler, K. Wuthrich, *J. Mol. Biol.* **1997**, *273*, 283-298.

[17] J. R. Maple, U. Dinur, A. T. Hagler, *Proc. Natl. Acad. Sci. U.S.A.* **1988**, *85*, 5350-5354.
